# Examining the Role of Community Engagement in Enhancing the Participation of Racial and Ethnic Minoritized Communities in Alzheimer’s Disease Clinical Trials; A Rapid Review

**DOI:** 10.14283/jpad.2024.149

**Published:** 2024-08-09

**Authors:** Sanaz Dabiri, R. Raman, J. Grooms, D. Molina-Henry

**Affiliations:** 1 Sol Price Schaeffer Center & Alzheimer’s Therapeutic Research Institute, University of Southern California, Los Angeles, USA; 2 Alzheimer’s Therapeutic Research Institute, University of Southern California, Los Angeles, USA; 3 Department of Economics, Howard University, Washington, DC, USA

**Keywords:** AD/ADRD clinical trial, recruitment, minoritized, disparities, community-based

## Abstract

**Background:**

Despite higher dementia prevalence in racial and ethnic minoritized communities, they are underrepresented in Alzheimer’s disease clinical trials. Community-based recruitment strategies are believed to yield positive outcomes in various fields, such as cancer and cardiovascular clinical trials, but their outcomes in Alzheimer’s disease and Related Dementias (AD/ADRD) require further study. In this systematic rapid review, we synthesized the available evidence on community-engaged recruitment strategies in enhancing participation in AD/ADRD clinical trials and observational study participation.

**Methods:**

We searched and identified studies describing a community-based recruitment approach for racial and ethnic minoritized communities across seven databases (Pubmed, OVID MEDLINE, Cochrane Central Register of Controlled Trials, CINAHL, PsychINFO, Web of Science, and EMBASE).

**Results:**

Out of 1915 screened studies, 49 met the inclusion criteria. Most studies employed multiple community-based recruitment approaches, including educational presentations, collaborations with community-based faith organizations, community advisory boards, and engagement with local clinics or health professionals. 52% of studies targeted more than one racial and ethnic minoritized population, primarily African Americans and then Hispanic/Latino. Gaps in knowledge about AD/ADRD, its increased risk among minoritized populations, distrust, and stigma were noted as barriers to research participation. Approximately 50% of the studies specified whether they evaluated their recruitment approaches, and in studies where approaches were evaluated, there was substantial heterogeneity in methods utilized.

**Conclusion:**

The quality of available evidence on the use of community-based recruitment approaches to include racial and ethnic minoritized populations in AD/ADRD research, particularly in clinical trials, is limited. Systematic assessment of recruitment strategies is urgently needed to increase the evidence base around community-engaged recruitment approaches.

**Electronic Supplementary Material:**

Supplementary material is available in the online version of this article at 10.14283/jpad.2024.149.

## Introduction

In 2021, the US Department of Health and Human Services published a National Plan to treat Alzheimer’s disease and related dementias effectively (AD/ADRD) by 2025 (1). Despite a growing focus on AD/ADRD clinical trials, recruitment of participants continues to be a challenge. Older adults participating in clinical trials tend to be non-Hispanic White, have a higher socioeconomic status, are married, or have a partner (2). In this systematic rapid review, we focused on community-based AD/ADRD recruitment strategies to engage racial and ethnic minoritized communities. We followed the US Census official race categories: White, Black/African American, Asian American, American Indian/Alaska Native, Native Hawaiian/Pacific Islander. In terms of ethnicity, individuals with shared cultural ties to Latin America are often identified as Hispanic, Latino, Latina, or Latinx. For the purpose of this paper, we refer to this group of individuals as Hispanic/Latino.

The risk and prevalence of dementia are higher among racial and ethnic minoritized groups, including African American/Black and Hispanic/Latino (3–5). Nevertheless, a systematic review of randomized controlled trials (RCTs) reported that racial and ethnic minoritized communities remain underrepresented in AD/ADRD clinical trials (6). In a series of 6 AD/ADRD cooperative trials, only 5% Hispanic and 6% African American participants were enrolled (7). Similar findings have been observed in the AD/ADRD cohort or observational studies (8). Currently, Hispanic/Latino individuals account for 52% of the population growth in the US (9), and this population is expected to increase by almost double from 63.6 million to 111 million by 2060 (9, 10). Moreover, it is estimated that by 2044 half of the US population will identify as belonging to racial and ethnic groups other than non-Hispanic White (11). To ensure the generalizability of AD/ADRD research findings, there is a greater need for the participation of racial and ethnic minoritized communities in AD/ADRD clinical trials and observational studies.

Recruitment challenges have been well-documented and can fall under individual, social/environmental, or broader economic, institutional, and cultural barriers. Some of the reported challenges are the participants’ reluctance stemming from the risk-benefit analysis of concerns around the intervention (12), the presence of comorbid conditions (13), and restrictive study protocols defined by specific inclusion and exclusion criteria (14–16). Lack of awareness of available clinical trials, primary care physicians’ lack of resources to refer patients (17), and participants’ distrust (12, 18, 19) can contribute to apprehension around participation. Moreover, lengthy trial duration (13), transportation challenges (20), caregiver or study partner burden (21, 22), socioeconomic status, participants’ specific unmet cultural needs, and differences in perceived risk for AD/ADRD (19, 23) are additional recruitment challenges. The barriers to participation may also vary depending on the specific racial and ethnic minoritized groups. While the majority of individuals from minoritized communities believe medical research to be biased against their communities (24), the distrust may be more pronounced among African American participants because of the historical instances of unethical research and abuse of power. A systematic review identified the legacy of the Tuskegee Study, institutional racism and discrimination, concerns about the research process, and disregard for cultural norms among research teams as distinct barriers for African American individuals to enroll in any health-related research (25). On the other hand, Hispanic/Latino participants in a focus group identified that using low-literacy recruitment material, bi-lingual staff, and informing that immigration status would not impact the research participation among Hispanic/Latino adults (26). Asian American communities are diverse in history, culture, and language; however, they are often grouped together in research. Barriers to participation among Asian American adults may be more specific to their social context, such as the level of acculturation or family support (25). For individuals identifying as Pacific Islanders, barriers may be attributed to cultural insensitivity and concerns about data use (25). Therefore, differences in barriers, unique attributes, and lived experiences of the different populations should be considered when recruiting for AD/ADRD clinical trials and observational studies. For instance, in the case of African American research participants, increasing trust, diverse representation in research teams, and acknowledging past abuses have been strategies to facilitate recruitment (26).

Community outreach and the engagement of community partners and stakeholders may serve as facilitators for enhancing racial and ethnic minoritized participation in AD/ADRD clinical trials and nonintervention types of research (27). Community-based organizations may have better access to and build relationships with potential study participants. Such efforts have been successful in the context of cardiovascular disease prevention (28–30) and cancer clinical trials (28, 31, 32). For instance, in a quest to identify the most successful recruitment strategy to engage African American communities in clinical trials, Otado et al. reviewed all clinical trials at the Howard University Clinical Research Unit. The 50 reviewed studies involved cognitive aging, sickle cell disease, HIV, posttraumatic stress disorder, genetics, hypertension and diabetes, cancer, stress, substance use, and alcohol research. In this study, anecdotal reports from study coordinators suggested that community outreach yielded the highest recruitment outcome (28). Notably, community engagement exists on a spectrum, with different levels of community involvement, ranging from an outreach with minimal community involvement to a shared leadership that is based on a bi-directional relationship between researchers and community members (33). In response to the 2018 initiative by the National Institute on Aging urging additional research at developing and evaluating diversity among AD/ADRD research participants (34), multiple reviews have synthesized the current understanding of recruitment and retention strategies of racial and ethnic minoritized groups into all AD/ADRD research (35, 36). These reviews reported on all types of recruitment strategies in AD/ADRD research. Despite this breadth of information, there remains a knowledge gap in differentiating recruitment efforts between clinical trials and observational studies in the AD/ADRD field. While progress has been made in enrolling racial and ethnic minoritized individuals in AD/ADRD research, challenges persist in clinical trials where the aim is to evaluate a pharmacological agent. Given that AD/ADRD clinical trials face unique recruitment challenges, the recruitment strategies may contrast with those used for observational studies, which typically involve less invasive procedures and a lower burden on participants and their study partners. In this systematic rapid review, we narrowed our focus to the utilization of community-based recruitment methodologies to enhance the representation of racial and ethnic minoritized populations in AD/ADRD research. By doing so, we aimed to offer an updated understanding of the current state of the community-based recruitment approach within the recruitment science, shedding light on its effectiveness and challenges among AD/ADRD clinical trials compared to observational studies. Additionally, we examined the current evidence on participants’ attitudes toward AD/ADRD and persistent barriers to recruitment in AD/ADRD research despite using community-engaged recruitment strategies. Our systematic review sought to answer the following question: Are there discernible differences in the type of community-based recruitment strategies used for AD/ADRD clinical engagement (clinical trials and studies that assess interest in clinical trial participation) and observational engagement (non-interventional studies and studies gauging interest in non-interventional AD/ADRD research? We further delved into differences in strategies across racial and ethnic minoritized communities, identified knowledge gaps in the recruitment science of AD/ADRD in clinical trials and observational studies, and discussed potential approaches to fill these gaps.

## Method

A rapid review is a knowledge synthesis approach that expedites the systematic review process by simplifying or omitting certain stages. This streamlined method allows for the timely and resource-efficient production of evidence synthesis. We conducted a rapid review of the literature using guidelines recommended by the Cochrane Handbook (37) over an eight-month period from 04/20/2023 to 12/12/2023. We registered the protocol of this rapid review in the International Prospective Register of Systematic Reviews (PROSPERO) database (ID: CRD42023427312) on 5/18/2023.

### Study selection

**Figure 1:**
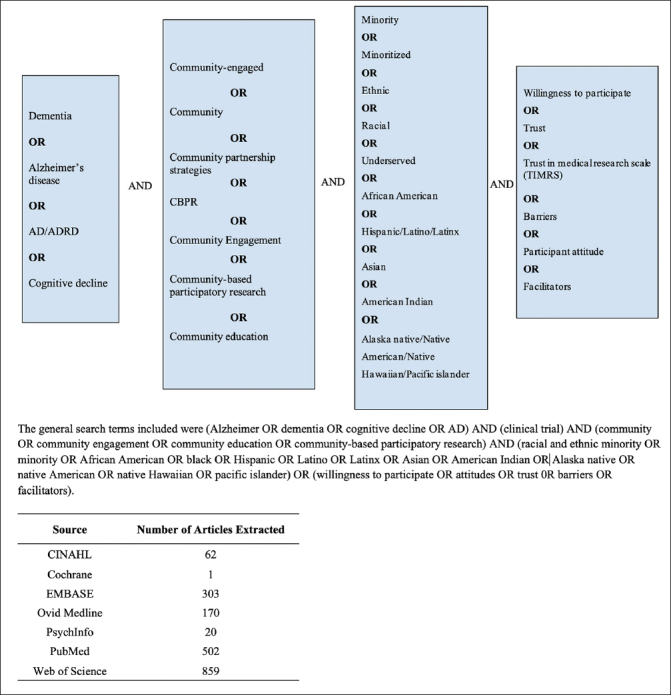
Search Terms

We searched across databases including: PubMed, OVID MEDLINE, Cochrane Central Register of Controlled Trials, CINAHL, PsychINFO, Web of Science, and EMBASE. We included any AD/ADRD-focused research published in English and a peer-reviewed journal that used at least one community-based recruitment approach to target participants from at least one racial and ethnic minoritized community (See Figure 1 for search terms and the number of articles extracted from each database). Community-based recruitment approaches were defined as any recruitment efforts that included community outreach that had a connection with community leaders, organizations, community church/faith-based groups, community health clinics, local businesses, community events, community senior centers, and community dementia services. Communities of focus were African American/Black, Hispanic/Latino, Asian/Asian American, American Indian or Alaska Natives, and Native Hawaiian or other Pacific Islander. Searches were not limited by time to ensure the capture of a more comprehensive understanding of community-based recruitment approaches and trace any emerging trends. Posters of conference abstracts were not included since the full description of the recruitment strategy would not be reported in the abstracts.

We included two types of published articles: 1. Studies that reported community-based recruitment efforts to increase racial and ethnic minoritized communities in AD/ADRD clinical trials or observational studies. 2. Recruitment studies that used community-based recruitment efforts to explore racial and ethnic minoritized communities’ attitudes and perceptions about AD/ADRD participation in clinical trials or observational studies.

A clinical trial, as defined by the NIH (38), is a research study where one or more human subjects are assigned to one or more interventions to assess the effects on health-related outcomes. In our review, we focused on community-based recruitment approaches in AD/ADRD research, including studies that assessed barriers and facilitators of clinical trial participation among racial and ethnic minoritized communities. We categorized both recruitment studies for AD/ADRD clinical trial engagement and AD/ADRD clinical trial studies together as Clinical Engagement. Observational studies, on the other hand, are those in which the investigator records observations and analyzes data without assigning participants to a specific intervention. These studies may focus on observing risk factors, natural history, or variations in disease progression and treatment without implementing an intervention (38). We grouped both observational studies and those gauging interest in non-interventional research participation together as Observational Engagement.

**Figure 2:**
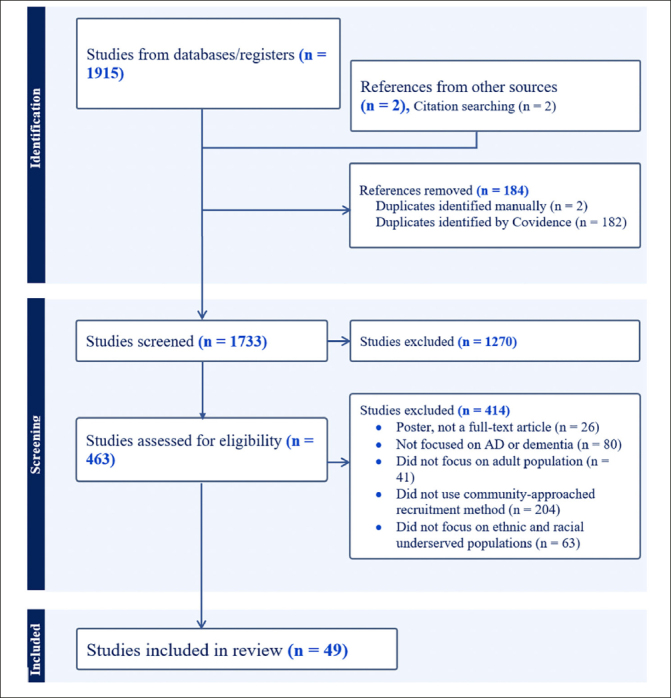
Flow diagram of search methodology for studies to include Studies were excluded if their target population did not specifically use a community-approached recruitment method to recruit racial and ethnic minoritized communities and if the study was not AD/ADRD related

References were imported to EndNote, and initial duplicates were removed. Electronic results were imported and screened using Covidence Systematic Review Software (2019). Duplicates were identified and excluded once the references were imported on Covidence. The consort diagram in Figure 2 details the screening and selection process of included and excluded studies. Studies were included if they described a recruitment approach that involved the community, and the focus of recruitment was on increasing participation of community members from different ethnic and racial groups. Furthermore, studies that included measures of knowledge of AD/ADRD, trust in the medical and research community, beliefs about AD/ADRD research, and willingness to participate were captured to summarize available evidence within the selected studies on participants’ attitudes and perceptions toward AD/ADRD clinical trials and observational studies.

### Data extraction

A single-reviewer data extraction approach was adopted in light of the limited time and resources available for this review. This decision aimed to warrant the timely completion of the review while maintaining a focus on the essential aspects of the evidence synthesis. To ensure the reliability and accuracy of the review, quality control measures were implemented, including the use of standardized data extraction and quality assessment tools, and periodic consultation with a secondary reviewer for critical stages. We adopted the data extraction item checklist in the Cochrane Handbook (37) for this review. Specifically, we extracted information regarding participants’ age, education, targeted minoritized population, sample size, study design, specific community-based recruitment approach, measures of participants’ perceptions and attitudes toward AD/ADRD research, inclusion/exclusion criteria, study aims, outcome variables, geographical location of recruitment, and key findings.

### Data quality assessment

Data quality was assessed using the Quality Assessment Tool for quantitative studies (39), which categorizes the assessment as weak, moderate, and strong based on eight criteria, including selection bias, study design, confounder, blinding, data collection methods, participant attrition, intervention integrity, and analysis. Joanna Briggs Institute Checklist for Qualitative Research (40) was used for qualitative and descriptive studies. Each item on the checklist helps the reviewer evaluate the extent to which the study meets established qualitative research standards, contributing to an overall assessment of the study’s reliability and validity. During the full-text screening, the quality of each article was assessed through all eight domains of the Quality Assessment Tool or the ten items of the Joanna Briggs Institute Checklist to check whether the items on the Qualitative Assessment Tool or Joanna Briggs Institute checklist were addressed in the articles.

## Results

The primary outcome of this review was to synthesize available evidence on differences in community-based recruitment strategies for AD/ADRD clinical trials versus observational studies. The secondary outcome was synthesizing available evidence on these strategies’ differences across racial and minoritized communities. Additionally, we explored participants’ attitudes and perceptions toward AD/ADRD research.

Our database searches yielded 1915 studies, and two additional studies were identified by cross-referencing. After removing duplicates, 1270 studies were excluded during title and abstract screening. A further 414 studies were excluded during full-text screening because they were either posters, lacked community-based recruitment methods, or were not focused on the AD/ADRD research. Additionally, articles lacking adult participants and articles that did not have an emphasis on recruiting racial and ethnic minoritized communities were also excluded (see Figure 2). We identified 49 studies that focused strictly on community-engaged recruitment strategies, emphasizing recruiting at least one racial and ethnic minoritized group (see Figure 2). Out of 49 studies, 11 focused on clinical trial recruitment or assessed willingness to participate in clinical trials (clinical engagement), and the rest of the articles were AD/ADRD observational studies (observational engagement).

### Characteristics of included studies

Ten studies (20%) utilized a qualitative approach (5, 41–49) without numeric outcomes, employing methods such as in-depth interviews, case studies, or written reports. Thirty studies (61%) used a quantitative approach (14, 50–78) with at least one numeric outcome. Five articles (10%) were descriptive studies, such as research protocols (79–83). There were four (8%) mixed-method studies (84–87). Of the 49 studies, 24 (49%) collected participants’ attitudes and beliefs about AD/ADRD and research participation. Thirty-nine (80%) studies used more than one recruitment approach. For instance, 21 studies (43%) participated in community events collaborating with community faith-based groups or local health professionals. Ten (20%) studies formed partnerships with a Community Advisory Board and another community organization, such as a church or a local health clinic, to create educational programs at churches, senior centers, and community events. Twenty-four studies (49%) reported a measure of participants’ attitudes and knowledge about AD/ADRD research. Fourteen studies (29%) identified barriers or facilitators to recruiting racial and ethnic minoritized populations, delineated by study participants (5 studies, 36%), community partners/liaisons (3 studies, 6%), or the investigators (6 studies, 12%). The characteristics of these studies are included in the following section and summarized in Table 1 and Table 2.

**Table 1:** Participants’ characteristics

**Author**	**Education**	**Age**	**Total number of participants: female, male**	**Racial/Ethnic Group: Sample Size**
Ashford 2022	16.4	57.2	Female 164 (82.8%)	AA Hispanic, other race
Arjouch 2020	75% more than HS in Arab American, 24% more than HS in Hispanic/Latino	51 in Arab American, 49 in Latinx	N (Arab American) = 183, 63.5% Female N (Latino) = 134, 76.9% Female	Middle-Eastern/Arab American Latino
Ashford 2021	14.3	57	N = 7353 Female 5871 Male 869	Latino 5662 658 non-Latino 148 decline, 885 unknown, 392 Native American, 77 AA, 54 Asian, 32 Pacific Islander, 3261 White,
Bachman 2009	12	73	N = 122 60% Female	AA(43.4%)
Ballard 1993	NR	NR	NR	150 Black not known for Hispanic
Bardach 2019	NR	3 groups: 76, 61.25, 59.38	N = 21 Groupl: 100% Female Group 2: 87.5% Female Group 3: 87.5% Female	AA
Bardach 2020	NR	78.8	88	AA
Barnes 2012	range(3–30)	72.8	N = 366 28.1% male	AA
Bleakley 2022	Did not complete HS 1.7% HS/GED10% Some college 18.3% Associate / technical degree 23.3% Bachelor’s degree 26.7% Graduate degree 20%	62.2	N = 60 30 Female 30 Male	Black 20 Hispanic 20 White 20
Boyd 2022	no degree/GED=55% >=2-yr college degree=45%	<40=46% 40–64=43% 65>=12%	N = 320 62% Female 37% Male	American Indian and Alaska Native
Chao 2011	12.6	68.4	N = 125 40.9% Male in outreach clinic 72.7% Male in community health fairs 56.5% Male in campus clinic 36.2% Male from other resources	Asian: Chinese American 125
Cocroft 2020	NR	NR	NR	AA
DeCaro 2022	NR	NR	N = 59 Sex NR	AA59
Epps 2015	NR	NR	N=33	AA33
Etkin 2012	52% had some college or more	61.6	N = 211 81% Female	AA24.8% Hispanic 24% Asian 6.2% White 65%
Fritsch 2006	13.8	74.1	N = 840 81.3% Female	AA134 White 680
Gallagher-Thompson, 2004	NR	NR	N=310 NR	195 Latino and 115 White
Gallagher-Thompson, 2006	NR	NR	N= 250 NR	116 Chinese American and 134 white
Gauthier, 1999	NR	NR	NR	AA
Han, 2021	less than Hs = 60%	81.5	50% Female	Korean American
Hinton, 2010	13	White: 77 AA:75 Hispanic:73 Other: 76	N = 404 51% Female White 71% AA Female 68% Hispanic Female	AA 145 Hispanic 124 White 51 Other 57
Howell 2016	Categories of education were presented for AA, Caucasian, and others. Over 70% had an Associate degree or higher	There were categories per race, but 53% were over 65 years	N = 218 98 Male	AA67 White 140 Other 11
Li 2016	12.79	73.9	N = 98 64 Female 34 Male	Chinese American
Lingler 2022	44.6% had some college or more, 45.6% had a university or postgraduate degree	47.4% were between 40 to 59 31.4% were 60–79 21.2% were 18–39	N=500 386 Female 114 Male	AA
Marquez 2022	High school (HS) & above: Older adults (43%), Adults (83%), Caregivers (62%), CBO (92%), Total HS and above: 62%	Adults: 32 Older adults: 67 Caregivers 56 CBO administrators:44 Total: 49 years	N= 181 69% Female	Latinos
McDougall 2010	13.4	75	N=265 77% Female	White 71.5% Black 11.5% Hispanic 16.9%
Meyer2020	14	60.91 caregivers 74.64 patients with dementia	N = 11 9 Female	Asian: Vietnamese
Milani 2021	education level (less than high school, at least high school or GED), 87.3% White, 79.1% nonHispanic Black, 82% Hispanic at least GED/HS	Age groups were broken down by race and ethnicity. 50–59: White (44.7%), Black (57.9%), Hispanic (40.2%), 60–69: White (34.9%), Black (32.4%), Hispanic (35.4%), 70–79: White (15.7%), Black (8.2%), Hispanic (18.3%), 80+: White (4.7%), Black (1.5%), Hispanic (6.1%)		
Neugroschl 2019	NR	74.4	N=49	Hispanic
Nkimbeng 2022	65% with a Bachelor’s degree or higher	60% were over the age of 55 years	N = 24 67% female	African immigrant community 79% were born in Liberia and were children of Liberian immigrants
Overman 2014	NR	NR	N = 19 16 Female, 3 Male	AA13 White 6
Parker 2022	NR	NR	NR	AA
Parveen 2018	NR	NR	N = 723 in Study 1 N = 1200 in Study 2	South Asian
Perales-Puchalt 2020	45% less than HS education	75	N=50 37 Female 13 Male	Hispanic
Portacolone 2020	55% some college and above	65	79% Female older adults, 80% caregivers, 82%admins, 79% total	AA134 Hispanic 4 More than 1 race or not specified 7
Rexroth 2010	NR	NR	NR	AA N not reported
Romero 2014	NR	68.2	74% Female	African America 30.9% White 65.9% Other ethnicities, including American Indian, Hispanic, Asian, or other 1.6%
Samus 2015	13.2	15% 90 yrs and older 60% 80–89 yrs 25% 70–79 yrs	N = 303 64% Female	AA29%
Sharma 2022	98.5% had at least HS education	NR	NR	AA, Latino, Asian American, and Indigenous communities No sample size is reported
Shaw 2022	College education (25.4%) High School (12.7%) Vocational/Trade (6.3%) Some Colleges (25.4%) Associate (17.5%) Bachelor (20.9%) Masters (15.9%) Doctoral (1.5%)	18–55:17.5% 56–65: 25.4% 66–75: 34.9% 76>: 22.2%	N= 66 90.5% Female	AA
Souder 2009	NR	NR	NR	Black N not reported
Sun 2014	Below 12th grade (32.2%) 12th grade or some college (31.4%) College graduate or above (36.4%)	72.43	N = 385 64.2% Female	Chinese American or Chinese immigrant
TaPark 2023	HS (37.9%) Some college (12.2%) Bachelor’s degree (27.8%) Master’s or higher (18%) Prefer not to say (4.1%)	53 (range 18 to 99)	N = 7040 4480 Female	394 Asian Indian 2359 ethnic Chinese 272 Filipino, 169 Japanese, 1242 Korean, 156 Native Hawaiians /Pacific Islanders, 2505 Vietnamese
Weiner 2023	16.4	55 to 90 yrs old	Goal is 51.30% Female	Underrepresented populations, including Black, Latino, Native American, Asian Recruitment is ongoing, no sample size was reported
Wiese 2021	11	70.4	N = 61 46 Female 15 Male	AA32 White 17 Afro Caribbean 7 Hispanic/Latino 3 Asian American 1
Williams 2011	NR	NR	N= 29	AA
Withers 2019	12	37 (range20–73)	73 Female 46 Male	86 Mexican and 37 Mexican-American family members
Zhou 2016	16.1 for AA 16.5 for white	72 for AA 73.9 for White	37 AA Female 51 white Female	AA47 White 78
O’Bryant 2014	Mexican American: 5.9 yrs in AD group, 6.6 in MCI group, 8.1 in NC group. NHW: 13.2 in AD group, 12.4 in MCI group, 14.3 in NC group.	Mexican American: 73.6 yrs for AD, 61.9 in MCI group, 58.7 in NC group. NHW: 79.4 for AD group, 74.4 for MCI group, 65.6 for NC group.	Total: 1069. Mexican American (N=435): 45% male for AD, 38% male for MCI, 29% for NC. NHW (N=633): 39% for AD, 33% for MQ, 32% for NC.	Mexican American NHW

• African American = AA, Non-Hispanic White = NHW

**Table 2:** Study characteristics

Author	Specific community engagement approach:	Measures of participant’s attitudes/knowledge about AD/ADRD	Study Aim	Facilitators to recruitment?	Barriers to recruitment?
Clinical Engagement^1^					
Bardach 2020	Health fairs and educational community symposiums Partnered with Garrett Davis to show 2 plays in collaboration with churches, community and senior centers, and senior living communities; and community online efforts, including distribution of event information through Facebook groups of AA churches and other predominantly AA groups and e-newsletters.	Willingness to participate in a clinical trial or longitudinal study	Explored whether attendance at AA community events encourages clinical trial and/or longitudinal research participation. Explored reasons event attendees do not participate in research.	Engagement in general audience outreach events	Too young to be included in a study, competing life demands such as work schedules or caring for a family member, lack of study partner, uncontrolled health conditions, general lack of interest
Etkin 2012	Partnered with faith-based organizations, senior centers, community educational presentations, local advertisement, and health fairs	NA	Described how strained and sedentary family caregivers of persons with AD were recruited into the Telephone Resources and Assistance for Caregivers (TRAC) study, a lifestyle physical activity clinical trial. Explored the enablers and barriers faced in recruiting and subsequently enrolling these family caregivers into this clinical trial.	NA	Possible conflict between the interests of academic institutions and provider agencies. Physicians were also difficult to contact. In larger medical centers, bureaucratic issues, and the potential of perceived competition made recruitment quite difficult. Adult day programs did not regularly meet with their families. Difficulty accessing caregivers. Facilities’ financial challenges. Some organizations no longer had sites or telephone numbers.
Marquez 2022	Partnered with local Community-based organizations (e.g., immigration, social, health-care services, advocacy organizations)	Knowledge about ADRD,	The overall goal of the Focus Groups was to understand the barriers and facilitators to participating in AD / ADRD research, including clinical trials.	Education about symptoms, prevention, and resources, use of Spanish language television, radio, social media, and health fairs. Use of endorsement from community organizations that are trusted by Latino communities. Altruism, researchers invested in physical presence in the community, free transportation, home visits, flexible schedules, and other incentives.	lack of knowledge of ADRD, distrust, fear of receiving a diagnosis, not being contacted, and participation in research are unheard of in their culture.
McDougall 2010	Partnered with Hispanic senior activity centers, churches, health fairs, and festivals. The intervention was implemented at seven sites in the community: four senior centers, a university-based wellness clinic, and two apartment complexes for low-income older adults	NA	Explored the effects of a memory training intervention on memory self-efficacy, metamemory anxiety, depression, and memory performance in a 5-year longitudinal study.	Culturally tailored cognitive instruments, adaptive performance testing, developing culturally appropriate health topics, addressing the cultural stigma of dementia, flexible scheduling, partnership with the Hispanic senior center to overcome mistrust,	Stigma, schedule conflict, mistrust
Meyer 2020	Partnered with the church, community health fairs, and Vietnamese doctor’s offices, attended the Lunar festival 20 min educational presentation	AD/ADRD knowledge	The pilot study used a single-arm, pretest-posttest design to examine the feasibility and acceptability of implementing the AD / ADRD caregiver intervention. An additional exploratory aim was to assess preliminary efficacy on caregivers’ AD knowledge and psychosocial outcomes	NA	NA
Overman 2014	Partnered with a local church and a local adult day center	NA	Devised a preliminary game-based intervention to improve the neuropsychological health of disadvantaged populations	NA	NA
Romero 2014	Regularly scheduled events included presentations at senior health fairs, family reunions, civic and senior groups, and educational venues at medical centers, senior housing facilities, lifelong learning groups, Duke University retiree associations, and churches	Interest in pharmacological prevention	Described coordinated efforts to create the Alzheimer’s Disease Prevention Registry (AD PR) of healthy volunteers who reflect the ethnically diverse local community. Interest in pharmacological prevention was assessed.	NA	NA
Samus 2015	Partnering with the Community Advisory Board and their community liaison (the Associated Jewish Federation of Baltimore) introduced the study team to its local network of 55 community partners.	NA	Provided a critical review of a multipronged recruitment approach used to identify, recruit, and enroll a diverse community-based sample of persons with memory disorders into an 18-month randomized, controlled dementia care coordination trial.	Expanding eligibility criteria such as geographic reach and inclusion of study partner	eligibility criteria
Shaw 2022	Partnered with the community church and health advocacy organization to provide a community education program	Knowledge, beliefs, and interest in clinical trial research	1) Explored how a culturally tailored community education program impacts clinical trial interest and enrollment in AD / ADRD research studies and 2) Identified how the African American community perceived the culturally tailored curriculum as applicable.	NA	NA
Withers 2019	Five presentations were conducted in healthcare centers or community halls	Cultural Beliefs about AD (CBAD) scale, interest in research participation	This mixed-methods study examines cultural beliefs about ADRD, genetic screening, and participation in research, including clinical trials, among at-risk Mexican-American participants.	NA	NA
Zhou 2016	Partnered with the community liaison who attended establishments such as senior centers and beauty salons, discussed the study, and distributed flyers, a caregiver support group. Provided community lectures	knowledge about AD, general attitudes toward research, perceived risk for AD, likelihood of enrolling in a clinical trial	Compared African American and White participants in their willingness to enroll in a hypothetical preclinical AD trial and examined barriers and facilitators in their decision-making	financial compensation, returning research results, community education	NA
Observational Engagement^2^					
Ashford 2022	Partnered with community professionals to create culturally tailored BHR enrollment campaigns for older Latino adults	Attitudes toward brain health research, reasons for joining BHR, continuing to participate, and barriers to participation	This study aimed to better understand online registry facilitators, barriers, and preferred communication channels of Black BHR participants.	understanding about own brain, impact on the community, better, technical support,	Lack of time or the research taking too long and burdensome Perceiving little to no value of the registry, or do not understand the value of the registry Technological issues Health issues
Ashford 2021	Partnered with community leaders to promote enrollment	NA	(1) to describe the CAL-BHR initiative, including how investigators developed and implemented the culturally tailored digital enrollment efforts, and (2) to report results from an interim feasibility analysis of the CAL-BHR culturally tailored digital enrollment efforts after lyear.	NA	A need to develop effective strategies to increase the enrollment of male Latino participants and Latino participants with lower education and to increase the completion of BHR tasks of enrolled participants.
Ajrouch 2020	Partnered with the Community Advisory Board, community organizations, and professional associations to provide regular health education series. For the Latino communities, a Latino Community Liaison was also hired	Motivation for attending	The Michigan Center for Contextual Factors in Alzheimer’s Disease (MCCFAD) engages with two underserved immigrant communities in Michigan – Middle Eastern/Arab Americans in metro-Detroit and Latinos in the Grand Rapids area – to recruit and retain two Participant Resource Pools (PRP)	Identify leaders (not only organizations). Clarify MCCFAD’s aims and goals. Rotate the location of community events. Arabic and Spanish translations/interpretations. Do not assume bilingualism.	Diversity (eg, socioeconomic and religious) Semantics/language use
Bachman 2009	Partnered with several community health clinics and physicians from rural and urban areas	NA	Presented the methodology utilized to create a predominantly AA cohort for the longitudinal study of risk factors in Alzheimer’s disease (AD). Identified geographically diverse clinical venues within South Carolina (SC), where large numbers of AA patients had already come to seek medical care.	NA	Skepticism about research in general, lack of AD acknowledgment in AA
Ballard 1993	Developed educational brochures to increase community awareness of AD / ADRD in general and of the CERAD research program in particular. Written at 6th-grade reading level in both English and Spanish. The brochures were distributed to senior centers, churches, nursing homes, local chapters of the Alzheimer’s Association, and other sites where Black and other minoritized communities are likely to come. Provided access to existing community resources for caregiver support, daycare facilities, and workshops. Forged relationships with local physicians.	NA	CERAD has taken various steps to reduce obstacles to recruiting blacks and Hispanics, including publishing educational brochures and encouraging active outreach programs for minority education and recruitment at nine sites.	Effective referral network involving the Black community, community education about AD, and professional staff sensitive to the needs of the black population.	Expenses, transportation difficulties, lack of rapport with clinic staff
Bardach 2019	Used three community advocates to recruit participants - researchers used a pain management support group, personal contact, and an existing relationship with the African American Dementia Outreach Partnership AADOP to recruit AA participants	Perception and influence of brain health	To better understand how African American participants conceptualize brain health and their ability to influence healthy brain aging.	NA	NA
Barnes 2012	Partnered with the Community Advisory Board, churches, subsidized senior housing facilities, retirement communities, African American clubs, organizations, fraternities and sororities, and social service centers	NA	Described the Minority Aging Research Study and the three cohort studies providing additional clinical and pathological data. Described efforts to obtain clinical and neuropathologic data in a cohort study of older African Americans without dementia, including recruitment and consenting procedures, maintaining high rates of follow-up participation, obtaining agreement for brain donation at the time of death, and achieving rapid autopsy.	NA	NA
Bleakley 2022	Recruitment efforts included recruitment drives in-person at community senior centers. Advertising in local newspapers and on Craigslist	Willingness to participate in brain health research studies, giving a DNA sample and cognitive tests every 6 months for a registry. Reasons behind participation	Presented results from semi-structured interviews conducted as the first step in a larger outreach message design study to increase enrollment of underrepresented groups in AD-focused participant recruitment registries. The interviews used the Reasoned Action Approach to understand what factors influenced participants’ decision to join an AD-focused participant recruitment registry.	Convenience, modality, providing written information, results transparency	Enrolling would be demanding, health problems, inconvenience, technology, transportation, having to travel, lack of information, medication side effect
Bleakley 2022	Recruitment efforts included recruitment drives in-person at community senior centers. Advertising in local newspapers and on Craigslist	Willingness to participate in brain health research studies, giving a DNA sample and cognitive tests every 6 months for a registry. Reasons behind participation	Presented results from semi-structured interviews conducted as the first step in a larger outreach message design study to increase enrollment of underrepresented groups in AD-focused participant recruitment registries. The interviews used the Reasoned Action Approach to understand what factors influenced participants’ decision to join an AD-focused participant recruitment registry.	Convenience, modality, providing written information, results transparency	Enrolling would be demanding, health problems, inconvenience, technology, transportation, having to travel, lack of information, medication side effect
Boyd 2022	Partnered with 2 community event organizers to provide health education and conduct surveys	Perception of AD, perceived risk in their communities, experience with AD, willingness to participate, preferred formats for communication	Surveyed AI/ANs in the Pacific Northwest to evaluate their perceived personal risk for AD, risk to their communities, levels of knowledge about AD, willingness to participate in AD research, and preferred formats for AD-related health communication.	NA	NA
Chao 2011	Partnered with community health fair organizers and community healthcare providers to provide educational presentations, research visits, and evaluations	Attitudes toward research, potential barriers to participation, motivation to participate	Described the results of efforts to recruit Asian Americans into longitudinal research in aging.	Getting more attention from physicians and better monitoring of cognition, monetary compensation, transportation compensation	No spare time, physical/reasons, and lack of transportation
Cocroft 2020	Partnered with community church to hold community health screenings, available resources, and recruitment outreach	NA	Discussed how the Alzheimer’s Disease Prevention Registry (ADPR) of the Joseph and Kathleen Bryan Alzheimer’s Disease Research Center at Duke University has been successful in achieving a racially diverse and research-ready cohort of cognitively healthy volunteers	Respectful and transparent community partnerships, personal relationships and responsiveness, sustainable funding sources	NA
DeCaro 2022	Partnered with community churches, community centers, community leaders, the Boston healthcare system, and the university to hold events on recruitment and updates on initiatives	The Jefferson Scale of Physician Empathy, the Dementia Attitudes scale, and the medical condition regard scale	The BU ADRC Ambassador Program had two separate aims: increasing student knowledge of ADRD through service learning and recruiting more Black participants in AD / ADRD research.	NA	NA
Epps 2015	Partnered with community leaders, faith-based leaders, local nurses, scientists, physicians, and counselors Health fairs	NA	Described the experience of a nurse researcher using culturally informed strategies to enhance recruitment in the African American population in southern Louisiana, as part of a study on family involvement in health promotion activities for older adults with dementia.	An analysis of the field notes revealed the salience of six themes, namely Gaining Trust, Visibility, Networking, Follow-up, Purposeful Activity, and Community Engagement.	Barriers that were overcome included a knowledge deficit about dementia in the target community and the cultural unsuitability of the terminology linked to dementia.
Fritsch 2006	Educational play Partnered with African American advisory board	Attitudes toward research willingness to participate knowledge about AD	Described their experiences in mounting 2 theater pieces geared toward African American audiences, with the goals of teaching important concepts about AD and encouraging audience members to consider participating in research studies of AD.	NA	NA
Gallagher-Thompson, 2004	Partnered with local health professionals	NA	Compared the participant retention rates of three different recruitment strategies in a sample of Latino and Caucasian family caregivers. Hypothesized that the “recruitment gap” would significantly close when using professional referrals, versus traditional recruitment strategies (i.e., media and non-professional referrals).	NA	NA
Gallagher-Thompson, 2006	Holding health fairs at community festivals Partnered with local health professionals	NA	Compared a consumer-centered approach with other relatively impersonal methods in the natural course of recruiting Chinese / Chinese American and white dementia family caregivers for participation in intervention research designed to evaluate strategies for decreasing distress. Three recruitment modalities were used for both ethnic groups: 1) media advertisements, 2) nonprofessional referrals (e.g., health fairs), and 3) professional referrals.	NA	NA
Gauthier, 1999	Partnered with community health center. Provided community education Sponsored community events and exercise groups in two senior complexes	NA	Discussed multiple trust-building strategies in the African-American community to recruit and retain participants for AD research	Develop trust within the community	Distrust of the medical establishment in Boston, fear of being used, and fear of being identified as having dementia or AD
Han, 2021	Partnered with the Community Advisory Committee to develop K-Plan. Provided cognitive screening and dementia literacy in the community, word-of-mouth, advertisements in ethnic newspapers, and referrals from a free community clinic for uninsured patients.	NA	(1) To assess the caregiver’s self-efficacy in obtaining medical services for dementia evaluation; and (2) to work with the dyad on strategies to manage identified barriers and provide individually tailored support and referrals for navigation assistance.	NA	NA
Hinton, 2010	Provided educational presentations at churches, senior centers, and support groups	NA	Described multifaceted strategy to establish an active outreach program and to proactively reduce key barriers to successfully recruit African American and Hispanic older adults into the University of California, Davis Alzheimer’s Disease Center (UCDADC). Identified variables that predicted whether or not older adults identified in a community-based screen would complete a comprehensive, research-quality clinical evaluation as a prerequisite for enrollment in a longitudinal research cohort.	Having bicultural and bilingual staff Education	Objections from adult children
Howell 2016	Community events and local primary care clinics	Knowledge of AD, Attitudes Toward AD	Examined the relationship between ADKS scores, demographic factors, and recruitment sources in a large multiracial cohort of older adults to determine predictors of ADKS scores. Furthermore, assessed participants’ attitudes toward AD to determine factors that influence attitudes toward AD, and whether there is a relationship between knowledge and attitudes toward AD	NA	NA
Li 2016	Partnered with community churches and senior centers to give lectures and evaluate potential participants	NA	Described and evaluated the rapid recruitment of elderly Chinese into clinical research at the Mount Sinai Alzheimer’s Disease Research Center (MSADRC).	Desires to understand own levels of cognitive health	Language barriers, but it was addressed
Lingler 2022	Community partner e-mail lists (including contacts at traditionally African American churches, sororities, fraternities, and community groups), through directed mailing service of individuals in the local community	Perceived AD risk, perceived benefit of research participation, perceived burden of research participation, trust in medical researchers	Examined factors associated with interest in AD research among Black or African American adults following exposure to RIDE narrative campaign materials	Building trust	lack of trust
Milani 2021	Community outreach at various locations, including recreational parks, local libraries, churches, laundromats, barbershops, public events, and community centers. Provided regular updates to the community on research findings and available resources	Past research participation, willingness to participate in different types of health studies, and their willingness to donate their brain for research	Compared the willingness of community members to participate in different types of health research by race and ethnicity using a sample of community members across Florida recruited by CHWs.	NA	NA
Neugroschl 2019	Partnered with the Community Advisory Board to develop a video intervention and play at community events	Attitudes, perception, and understanding of normal aging and memory, and knowledge about dementia	Used community events to assess whether watching the video had an effect on attendees’ interest in obtaining a memory screening	NA	NA
Nkimbeng 2022	Partnered with the Community Advisory Board to provide educational presentations and recruit participants	Dementia knowledge (DKAS), barriers to accessing care, attitudes toward dementia and mental health	(1) Develop a culturally informed community conversation guide that guides community conversations (focus groups) about dementia care and access, and (2) use the qualitative data to design and administer a dementia care needs and resources survey with the community.	NA	NA
O’Bryant 2014	Used CBPR approach: Partnered with the local hospitals and clinics (including multiple neurology clinics and Federally Qualified Health Centers [FQHCs]) as well as senior citizens’ organizations. Presented information at community events, churches, and food banks, as well as through door-to-door solicitation and clinic-based recruitment.	NA	To provide a characterization of Mexican Americans with MCI and AD. When compared to non-Hispanic whites, Mexican Americans with AD and MCI would (1) be younger, (2) have poorer global cognition and increased disease severity, (3) have higher rates of diabetes and depression, and (4) express a lower frequency of the ApoEe4 allele.	NA	NA
Parker 2022	Partnered with community dementia support groups and ADS	NA	Proposed three key constructs of Critical Race Theory to provide a useful framework for informing recruitment and enrollment of Black AD / ADRD caregivers. The framework can be used in conjunction with national initiatives to recruit Black caregivers of people living with dementia into dementia-care research.	NA	NA
Parveen 2018	Adopted a person-centered approach by involving patients, caregivers, members of the public, and support workers, including South Asian panel members	NA	The Caregiving HOPE study aimed to involve experts by experience (caregivers, people living with dementia, members of the public, and support workers) using a person-centered approach to involvement.	Personal factors of PPI such as ability, potential, and sense of well-being. Build a relationship with the I to increase knowledge of AD and trust in the PI. Person-centered philosophy. Diverse representation among the panel members	Funding, translation cost for members
Perales-Puchalt 2020	Partnered with senior centers, along with the Community Advisory Board, Hispanic caregiver advocates, and national Hispanic leaders to hold three educational presentations	ADRD knowledge, interest in participating in ADRD pre and post-survey	To assess the preliminary efficacy of a recruitment educational strategy among older Hispanics on ADRD knowledge, research participation attitudes, and enrollment. Used different outcomes to assess the strategy’s preliminary efficacy, including self-reports and metrics.	NA	NA
Portacolone 2020	Partnered with local community-based organizations serving African American communities	Drivers of trust	Examined factors that influence participation in dementia research among African American older adults and caregivers of African American older adults with dementia, with an emphasis on understanding factors related to trust. Examined expectations associated with trust overall, as well as expectations associated with trust toward researchers and community-based organizations.	Expectations associated with gaining trust among African American older adults included being caring, reliable, useful, and established relationships. The use of easy-to-comprehend research materials and consistency of involvement and support of the community. Representation of African American research team members and leaders of science.	distrust
Rexroth 2010	Partnered with churches, community centers, and senior centers to run AD presentations. Included lunch and used an African American RN to run the education meetings.	NA	Discussed the successes and challenges of reaching into African American communities and the work conducted in Indianapolis	Conducting research within the community, representation of the target population within the research teams, and providing meals. Developing relationships with community leaders over time.	Challenges due to low health literacy..
Sharma 2022	Partnered with the Alzheimer’s Association and other local organizations. Utilized prior relationships with staff from retirement communities with graduated levels of care to identify potential participants, disseminate information about the study, and promote snowball recruitment. Hired a Latino research assistant to assist with recruitment.	NA	Described how they adapted standard in-person participant recruitment and qualitative data collection methods for virtual use in a study of decision-making experiences in older adults with AD / ADRD	NA	Language barrier
Souder 2009	Partnered with the Black Advisory Board to develop a lay educator approach for AD presentations	NA	Adopted a lay educator (LE) approach to bridge the gap between the community and university-based research center. As a result, the LE program contributed to a significant increase in the number of AD presentations given to the Black community.	NA	The informational packet contained an overwhelming amount of information. The control participants were required to bring a family member to the initial interview.
Sun 2014	Chinese senior centers, church groups,	Knowledge of AD. Experience, cultural beliefs of AD, and resource variables	Examined knowledge of Alzheimer’s disease (AD) and correlates of AD knowledge in a sample of Chinese American older adults living in the Phoenix metropolitan area of the United States.	NA	NA
TaPark 2023	Close collaborations with community partners, including the National Asian Pacific Center on Aging (NAPCA, a CARE community partner), regular community outreach events were organized and provided education on health topics	NA	Described the development, implementation, and early results of the CARE Registry. Increased AAPIs’ participation in ADRD research by applying principles of the community-based participatory research (CBPR) approach to the development and sustainment of CARE, including forming a collaborative team of “trusted sources of information.”	NA	NA
Weiner 2023	Recruitment strategies were guided by a Community-Science Partnership Board and developed by the Diversity Task Force. Research methods were culturally appropriate	NA	ADNI4 will increase URP enrollment via community-engaged strategies and concomitantly deploy culturally informed assessment and research methods (e.g., new sociocultural measures, loosening exclusion criteria). It will use innovative technologies such as remote digital cognitive assessments and ultra-sensitive plasma assays for AD biomarkers that overcome the shortcomings of current CSF and PET assessments to help select participants for in-clinic studies and to monitor longitudinal progression.	NA	NA
Wiese 2021	Partnered with the Community Advisory Board and community church to hold educational presentations and recruitment	The Basic Knowledge of Alzheimer’s Disease (BKAD) survey assessed AD knowledge,	Evaluated the utility of using the telephone to conduct clinical, faith-based research with rural, underserved participants. To determine if providing ADRD information would be useful for increasing basic AD knowledge, identifying heightened AD risk, and increasing provider referrals when indicated in a small, rural, older, ethnically diverse, and largely farmworker (58%) cohort	NA	NA
Williams 2011	41 health fairs, 81 AD presentations, 36 health provider training	NA	The African American Outreach (Satellite) provided educational outreach to facilitate African American recruitment for longitudinal studies at the Washington University Alzheimer’s Disease Research Center (ADRC). Described the Satellite’s recruitment methods, plan for community engagement, results of recruitment efforts, and potential for replication	NA	NA

1. Clinical trials or studies that assessed participants’ attitudes or perceptions regarding AD/ADRD clinical trial participation; 2. Non-Clinical Trials/Observational Studies/Descriptive Studies or studies that assessed participants’ attitudes or perceptions regarding AD / ADRD research participation

## Data synthesis

### Geographical Location

The locations of the studies varied. Eleven studies (22%) did not disclose the location of recruitment and enrollment of sample populations. We found only one study in the UK (46) that focused on racial and ethnic minoritized communities outside of the US. The distribution varied widely, with a large portion of the studies (30%) conducted in California. Some places were in major urban centers such as New York City, Los Angeles, and Chicago), while others were in a mix of urban and rural areas (Kentucky, central Texas). Only six studies (12%) described the location of their recruitment efforts as rural areas, with 80% reporting the community church as their leading community partner (43, 73, 76, 78) to engage rural communities. In addition to this partnership, studies in rural areas used educational programs (73, 76, 86, 78) as community outreach, forged more relationships with local health professionals (78, 43) for referrals, and participated in health fairs (78,43). Among studies that described their recruitment location as urban (32 studies, 65%), 55% participated in health fairs and other community events, 45% used education as an outreach strategy, 33% partnered with local health professionals and local health clinics, and 30% formed relationships with community churches, and 24% developed their community-based recruitment strategies in partnership with their Community Advisory Boards. Locations in the Intermountain West and part of the Midwest of the USA were not represented among the studies reviewed. The approximate area of recruitment efforts is available in the supplemental file, indicating where studies reported their geographic locations.

Characteristics of studies target populations

Of 49 studies, 23 (47%) focused on more than one racial and ethnic minoritized population, with 13 studies (23%) also recruiting non-minoritized individuals (Non-Hispanic White adults). Most studies reported on recruiting African American adults in both clinical (8 studies, 73%) and observational engagement (20 studies, 53%) categories. Three studies (27%) reported on Hispanic/Latino adults in clinical engagement, and 12 studies (32%) in observational engagement. Two studies (18%) reported on Asian participants in clinical engagement, and nine studies (24%) in observational engagement. Five studies (13%) reported on Native American/Alaskan Native or Pacific Islander populations among observational engagement.

Thirty studies (61%) reported that most participants identified as female. Eight studies (16%) did not report the sex of the participants. Overall, we observed that aside from race and ethnicity, other attributes of the target populations, such as education, age, sex, occupation, and income, were not consistently described. Only 31 studies (63%) reported educational attainment and 34 studies (69%) reported their participants’ age/age group. Table 1 summarizes the characteristics of the sample populations.

### Community-based recruitment strategies

Recruitment strategies were variable, but 21 studies (43%) used community outreach in the form of educational presentations to raise community awareness and recruit participants. Twenty-one studies (43%) used different types of community events, including health fairs (3, 41, 83, 43, 50, 49, 51, 56, 58, 60, 62, 71, 74), cultural events (55, 63, 50, 66), and community fairs (47, 48, 81, 71), to raise awareness and enroll participants. Thirteen studies (27%) reported partnering with the local clinic, such as free community clinics (61), neurology clinics and Federally Qualified Health Centers (78), local wellness clinics (50), neighborhood health centers (81, 79, 63), community health professionals (49, 72, 3, 43, 79, 56), local chapter of Alzheimer’s Association, Alzheimer’s Disease Research Center (ADRC; 83), and other local healthcare agencies (60) as another commonly reported recruitment approach. Ten studies (20%) partnered with a local Community Advisory Board to develop recruitment material or to disseminate information. Among partner organizations, study investigators engaged with local businesses, community care programs, community liaisons, African American clubs, community support groups, senior centers, local media, and libraries. A large proportion of the studies (16 studies, 33%) described partnering with community faith-based organizations to disseminate study materials, hold educational presentations or identify potential participants.

Among our 11 reviewed articles in the clinical engagement category, five implemented a nonpharmaceutical intervention (e.g., lifestyle physical activity (58), cognitive training (68, 50)) that also included caregivers as their target populations. For instance, McDougall (50) partnered with Hispanic senior activity centers, churches, health fairs, and festivals to increase African American and Hispanic participation. At seven community sites, they randomly assigned participants to either a self-efficacy memory intervention or a comparison group with structured lectures on health improvements. In another study, Overman et al. (68) collaborated with a local church and adult center to focus on African American recruitment. They tested a game-based intervention to improve the neuropsychological health of older adults. Etkin et al. (58) implemented community participatory research and partnered with a faith-based and other senior service organization to increase the number of African American, Hispanic/Latino, and Asian American participants in their caregiver lifestyle physical activity clinical trial. Meyer et al. (66) collaborated with a local church and Vietnamese physicians, actively engaging in health fairs and Lunar festivals to host educational presentations aimed at recruiting Vietnamese American participants. Their objective was to implement a single-arm pretest-posttest design to test a psychosocial intervention for Vietnamese American caregivers of individuals with AD/ADRD. Samus et al. (72) analyzed a multifaceted community-based recruitment approach involving gatekeepers and community outreach to enroll racial and ethnic minoritized populations into an 18-month randomized controlled dementia care coordination trial. The rest of the clinical engagement studies assessed attitudes and willingness toward clinical trial participation. For instance, Romero et al. (71) held community events for educational presentations at health fairs, family reunions, civic groups, medical centers, and senior housing facilities to increase African American participants in the ADPR registry and assessed participants’ interest in a pharmacological prevention trial.

As described in the previously mentioned articles, the majority of clinical engagement studies (10 studies, 90%) employed multiple community-based recruitment strategies. Participating in community events emerged as the most frequently used approach (7 studies, 64%), followed by community church partnership (5 studies, 45%), educational presentations (5 studies, 45%), collaboration with local health professionals and health clinics (3 studies, 27%) and engagement with Community Advisory Boards (1 study, 9%). In contrast, studies in the observational engagement category prioritized educational presentations (15 studies, 39%) and participating in community events (15 studies, 39%), followed by collaboration with community churches (11 studies, 29%), health professionals or community health clinics (11 studies, 29%), and community advisory boards (9 studies, 24%).

Table 2 lists specific community-engaged recruitment strategies for each study. Recruitment strategies across racial and ethnic groups exhibited a high degree of consistency, indicating minimal variation among the groups. However, among the reviewed observational studies, recruitment materials were developed in different languages, such as Chinese (56, 85, 60, 64), Spanish (14, 51, 76, 80, 62, 70), and Arabic (51), to recruit Asian American, Hispanic/Latino, and Middle-Eastern participants. Including bilingual research team members (45, 64, 85) was another approach specific to engaging Hispanic/Latino and Asian American participants.

### Recruitment approach evaluation

Twenty-five studies (51%) reported the impact of their recruitment approaches. However, the evaluation method or the specification of a “successful” recruitment approach was not consistent across the clinical or observational engagement studies. Overall, only six studies (12%) compared different recruitment methods to report which approach yielded the highest number of enrollments. Compared to traditional recruitment strategies such as media and non-professional referrals, forming collaborative relationships with community healthcare agencies showed an increase in the recruitment of Hispanic/Latino (14) and Chinese American (60) individuals. Meyer et al. (66) compared different outreach efforts, such as community partner agencies, community festivals, community presentations, and other types of effort, such as word-of-mouth or referrals. Their findings showed community partner agencies produced the majority of study referrals (66). Zhou et al. (77) compared different recruitment outcomes from community talk, ADRC Registry, community liaison, community referrals, Banner Alzheimer’s Prevention Initiative Registry, caregiver support program, and clinical referrals, and found that through community liaison, they were able to recruit more African American participants. Samus et al. (72) compared five recruitment strategies: community liaison, community organizations that either sent letters about the study or distributed study materials, Johns Hopkins dementia research registries, and general community outreach. Their findings showed that the majority of African-American participants were referred by their community liaison. Thirteen studies (26%) monitored how many racial and ethnic minoritized participants they recruited and contributed to the success of the recruitment strategy. Finally, six studies (12%) reported that they either met their recruitment target or surpassed their goal.

AD/ADRD clinical trial recruitment has rigorous inclusion criteria compared to AD/ADRD observational research. Therefore, recruitment approaches that are successful in engaging racial and ethnic minoritized communities may not necessarily work in AD/ADRD clinical trials. However, the limited evidence that we reviewed demonstrated that through multiple community engagements with community liaisons such as community churches and community health clinics, and attending community events such as health fairs, researchers met/surpassed their target racial and ethnic recruitment goal (58, 72) or they evaluated community-engaged recruitment method by comparing their outreach with other types of recruitment approaches (53, 66, 71).

Most studies did not describe using theory to guide their recruitment strategies. Eight studies (16%) reported using frameworks with observable patterns, such as adopting recruitment approaches that are culturally sensitive, engaging the community, and having tailored messaging. Among clinical engagement studies, Samus et al. (72) crafted a recruitment methodology anchored in community-based participatory research (88) and gatekeeper outreach models (89), which cultivated active participation and trust within local communities. Etkin et al. (58) combined social marketing principles with community-based participatory research (88), utilizing precise messaging and community engagement to improve recruitment efforts. Shaw et al. (73) devised a culturally-tuned educational program through the Cultural Accommodation Model, recognizing elements such as religion, spirituality, and diet as key to involving African American communities.

Among observational engagement studies, Bleakley et al. (5) adopted the Reasoned Action Approach ((RAA (90)) to identify cognitive and motivational determinants that affect individuals’ decisions to participate in AD/ADRD studies. Wiese et al. (76) used the faith-based participatory model (FMM) to involve religious leaders in shaping programs to fit the congregation’s needs and values. They also trained community members to serve as faith-based health educators and utilized local pictures, resources, and language to ensure the material was culturally appropriate. Parker et al. (69) implemented Critical Race Theory in devising recruitment methods for African American/Black caregivers of individuals with AD/ADRD, aiming to counteract systemic racial inequities in healthcare by using culturally appropriate and customizable recruitment materials to reach and recruit racial and ethnic minoritized communities. Sun (85) applied the Explanatory Model (91) and the Common-Sense Model (92) to gain insights into how individuals perceive disease, blending lay health education and cultural considerations into recruitment practices. Lastly, Parveen et al. (46) based their recruitment strategy on the concept of patient and public involvement, proposed by Arnstein (93) and Morrow et al. (94), ensuring that collaboration with caregivers, those with dementia, and community experts was central.

Moreover, only a few clinical (44, 58, 66, 71, 72, 77) and observational studies (14, 39, 45, 55, 56, 59, 66, 78, 80, 96, 97) reported whether the relationships researchers formed with the community were new or existing prior to their research projects. However, the number of articles with existing community partnerships (11 studies, 22%) exceeded the number of newly formed relationships (7 studies, 14%).

### Participants’ attitudes and perceptions

Out of 24 studies (49%) that measured participants’ attitudes toward dementia and AD/ADRD research, five (20%) were clinical engagement studies (44, 53, 66, 71, 73). The specific assessments were the basic knowledge of AD (BKAD) (96), 30-item Alzheimer’s disease knowledge scale (ADKS) (97), 13-item epidemiology/etiology disease scale (EDS) (98), dementia knowledge (DKAS)(99), barriers to accessing care, drivers of trust, trust in medical researchers, perceived risk for AD/ADRD, perceived benefits from participation, cultural beliefs about AD (CBAD)(86), willingness to participate, willingness to donate brain for research, motivation to participate, and preferred formats for communication. Many of the elicited attitudes toward dementia in all racial and ethnic minoritized groups suggested that knowledge of AD was lacking (43, 44, 55, 63, 82, 84–86, 70, 77). In contrast to non-Hispanic White individuals, racial and ethnic minoritized communities exhibited a lower perceived risk of AD/ADRD. For example, only half of AI/AN participants thought AD/ADRD was a major health problem among AI/ANs, while 77% of the participants viewed AD/ADRD as a major health problem for the general public (55). In older Chinese American adults, being male and having lower education levels were related to less AD/ADRD knowledge (85). Similarly, African American participants had a lower AD/ADRD knowledge compared to White participants, and AD knowledge was also associated with education and attitudes toward AD/ADRD prevention and treatment (63). Hispanic/Latino participants also reported a lack of knowledge, fear of receiving a diagnosis, and cultural stigma regarding research participation as their barriers to joining either an observational (52, 62) or clinical trial study (44).

As expected, distrust of the research community (47, 65, 79, 81, 95), knowledge deficits (5, 43, 79, 82, 52, 44), and logistical issues such as transportation, schedule conflict, or lack of time were among the highest reported barriers in the studies (5, 41, 56, 80, 46, 87, 95). Other widely cited barriers were stigma (86, 95), health challenges (5, 41, 56, 52), and language barriers (48, 51, 64). Barriers specific to reviewed clinical trial studies from the research teams’ perspectives included difficulties in engaging physicians, bureaucratic hurdles in larger medical centers, reliance on adult day program staff for family contact, working with distant caregivers, and financial constraints hindering agency support (58). Among observational studies, researchers reported objections from adult children and their decision to veto parents’ enrollment in the study (62).

Among the observational and clinical trial articles, some barriers were addressed by attempts to overcome the broader institutional and cultural issues. Engaging in strategies to improve trust appeared to be the most important facilitator, especially for African American participants. In a qualitative study (47), African American participants suggested researchers and academic institutions invest in the health and well-being of African American communities, involve African American researchers in research teams, and enhance information sharing between research institutions and African American communities to gain their trust. Community education and enhancing AD/ADRD knowledge by increasing diverse representation in the research teams, addressing community needs, and using culturally appropriate and easy-to-comprehend recruitment materials also seemed to facilitate the recruitment approach. Ballard et al. (1993) (80) reported that involving the African American community, community education about AD/ADRD, and research teams being sensitive to the needs of the African American community were the key facilitators of increasing enrollment over a two-year period of active recruitment. Similarly, McDougall et al. (2010) (95) used culturally tailored cognitive instruments, culturally appropriate health topics, reducing the cultural stigma of dementia, adaptive performance testing, and scheduling as essential facilitating strategies to overcome mistrust and increase Hispanic/Latino and African American participation. Table 2 lists all barriers and facilitators that were reported in the studies.

### Data Quality

The quality of studies varied. We used the Joanna Briggs Institute checklist to appraise the methodological quality of qualitative or descriptive studies. All studies stated the research methodology and objectives, represented participants’ voices, had evidence of ethical approval, and conclusions appeared to be based on analysis or interpretation of the data. Out of 14 studies (29%), two met all ten quality criteria. Six studies (12%) met nine criteria, two (4%) met eight criteria, and the remaining four (8%) met at least four criteria. The majority of articles lacked clarity regarding the researchers’ cultural or theoretical orientations and did not acknowledge the potential bidirectional influence between the researchers and their studies. Additionally, there was a discrepancy between the selected research methodologies and the representation of the analyses (see Table 3).

**Table 3:** Joanna Briggs Institute Checklist for Qualitative Research Results

**Author**	**Philosophical Perspective**	**Research Objectives**	**Data Collection Methods**	**Representation & Data analysis**	**Interpretation**	**Cultural & theoretical perspective**	**Researcher’s Influence**	**Participants’ voices**	**Ethics**	**Flow**
Bardach 2019	Y	Y	Y	Y	Y	Y	Y	Y	Y	Y
Bleakley 2022	Y	Y	Y	Y	Y	Y	U	Y	Y	Y
Cocroft 2020	Y	Y	U	U	U	Y	U	Y	Y	Y
Epps 2015	Y	Y	Y	Y	Y	N	N	Y	Y	Y
Marquez 2022	Y	Y	Y	Y	Y	Y	U	Y	Y	Y
Neugroschl 2019	Y	Y	Y	Y	Y	Y	N	Y	Y	Y
Portacolone 2020	Y	Y	Y	Y	Y	Y	N	Y	Y	Y
Sharma 2022	Y	Y	N	N	N	Y	N	Y	Y	Y
Souder 2009	Y	Y	Y	Y	Y	Y	Y	Y	Y	Y
Ballard 1993	Y	Y	U	Y	Y	N	N	Y	Y	Y
Gauthier, 1999	Y	Y	Y	U	Y	Y	Y	Y	Y	Y
Parveen 2018	Y	Y	Y	N	Y	N	N	Y	Y	Y
Rexroth 2010	Y	Y	U	Y	Y	Y	N	Y	Y	Y
Williams 2011	Y	Y	Y	Y	Y	Y	N	Y	Y	Y

Y= Yes, N= No, U= Unclear; Philosophical Perspective: Is there congruity between the stated philosophical perspective and the research methodology? Research Objectives: Is there congruity between the research methodology and the research question or objectives?; Data Collection Methods: Is there congruity between the research methodology and the methods used to collect data?; Representation & Data analysis: Is there congruity between the research methodology and the representation and analysis of data?; Interpretation: Is there congruity between the research methodology and the interpretation of results?; Cultural & theoretical perspective: Is there a statement locating the researcher culturally or theoretically?; Researcher’s Influence: Is the influence of the researcher on the research, and vice-versa, addressed?; Participants’ voices: Are participants, and their voices, adequately represented?; Ethics: Is the research ethical according to current criteria or, for recent studies, and is there evidence of ethical approval by an appropriate body?; Flow: Do the conclusions drawn in the research report flow from the analysis, or interpretation, of the data?

We utilized the Quality Assessment Tool for quantitative studies. The majority of 36 quantitative studies received a «weak» rating due to high risk of bias, including selection bias (n = 20, 56%), study design (n = 9, 25%), confounders (n = 26, 72%), data collection method (n = 10, 28%), withdrawal and dropouts (n = 14, 39%), intervention integrity (n = 7, 19%), and analysis (n= 12, 33%). Tables 3 and 4 demonstrate the results of quality assessments.

**Table 4:** Quality assessment of quantitative studies

**Author**	**Selection Bias**	**Study Design**	**Confounder**	**Blinding**	**Data Collection Methods**	**Withdrawal and Dropouts**	**Intervention Integrity**	**Analysis**
Ashford 2022	W	M	W	NA	M	M	NA	S
Ajrouch 2020	M	M	W	NA	M	W	NA	W
Ashford 2021	M	M	W	NA	M	M	NA	W
Bardach 2020	M	M	W	NA	M	W	NA	W
Barnes 2012	W	S	S	NA	S	S	NA	M
Boyd 2022	M	M	S	NA	S	W	NA	S
Chao 2011	M	M	W	NA	M	W	NA	W
DeCaro 2022	W	M	W	NA	W	W	W	M
Etkin 2012	S	S	W	NA	S	S	M	W
Fritsch 2006	W	M	W	NA	M	M	NA	M
Gallagher-Thompson 2004	M	W	W	NA	W	M	NA	M
Gallagher-Thompson 2006	M	W	W	NA	W	M	NA	M
Han 2021	W	M	W	NA	S	S	M	M
Hinton 2010	M	S	W	NA	M	M	NA	M
Howell 2016	W	M	S	NA	W	M	W	S
Li 2016	W	W	W	NA	W	W	NA	W
Lingler 2022	W	W	W	NA	M	W	NA	M
Meyer 2020	W	M	W	NA	M	M	M	M
Milani 2021	W	W	M	NA	W	W	NA	M
Nkimbeng 2022	W	W	W	NA	W	W	NA	W
Overman 2014	W	M	W	NA	M	S	W	M
Parker 2022	S	M	W	NA	NA	S	NA	W
Perales-Puchalt 2020	W	M	W	NA	M	M	W	M
Romero 2014	M	W	W	NA	W	W	W	M
Samus 2015	W	M	W	NA	M	M	M	W
Shaw 2022	W	M	M	NA	M	M	W	M
Striley 2019				NA				
Sun 2014	M	M	M	NA	M	M	NA	S
TaPark 2023	M	M	W	NA	M	M	NA	W
Weiner 2023	W	M	NA	NA	M	NA	NA	NA
Wiese 2021	M	M	M	NA	W	M	W	M
Williams 2011	W	M	W	NA	W	W	NA	W
Withers 2019	W	W	W	NA	M	W	NA	W
Zhou 2016	W	M	M	NA	M	W	NA	M
Bachman 2009	S	S	W	S	M	M	NA	S
O’Bryant 2014	W	W	M	NA	M	W	NA	M
McDougall 2010	S	M	W	W	S	M	M	M

Strong = S, Moderate = M, Weak = W

## Discussion

Our rapid review suggested that strategies were variable among clinical trials and observational studies that utilized community-based recruitment approaches. Efforts to increase AD/ADRD awareness and recruitment outcomes by participating in community events, such as health fairs, were predominantly used in clinical trials. Whereas, efforts aimed at enhancing AD/ADRD knowledge through providing community educational presentations and participating in community events were equally employed by observational studies. Most of the included studies formed alliances with community faith-based organizations and some with community health professionals to recruit racial and ethnic minoritized populations. However, the limited evidence that we reviewed suggested that clinical trials did not report establishing relationships with local health professionals/health clinics as frequently as they did with faith-based organizations. Indeed, difficuly in engaging with community physicians was one of the noted barriers in our reviewed studies. Collaboration with local healthcare providers can enhance clinical trial awareness and may also reduce the distrust in pharmacological interventions if the referrals come from local physicians with existing relationships with the community.

Evidence of recruitment success can be challenging when using a community-based approach, as most studies can conduct multiple concurrent recruitment strategies. Therefore, teasing which strategy yielded the best recruitment outcome may be difficult. The absence of a “best” single strategy suggests that a multifaceted approach to recruitment, incorporating various strategies, may be more appropriate. Approximately half of the reviewed studies evaluated their recruitment strategies, with varying evaluation methods reported for “successful” community-based approaches. Comparison of recruitment methods for enrollment rates was limited. Monitoring numbers of recruitment of racial and ethnic minoritized participants was more common as a metric of success, and several studies reported they met or exceeded their recruitment targets. A commonality observed among these studies was the utilization of multiple recruitment strategies. Rigorous randomized community-based recruitment strategies should compare and identify the most successful approach to recruiting racial and ethnic minoritized communities in AD/ADRD clinical trials. Community-based approaches require building relationships between the research team and community members, which requires time and effort. However, studies are mandated by sponsors to submit summary results within a certain time period. Given the fact that community-based recruitment outcomes may not immediately occur, this becomes specifically challenging for clinical trial studies as they have enrollment timelines, and thus, evaluating what efforts encouraged participation in a rigorous way becomes additionally burdensome.

Multiple studies reported utilizing existing or new relationships within the community they were attempting to recruit. However, most studies did not explicitly describe whether their relationship with their focus community organizations or gatekeepers was new or whether they had an existing rapport. Understanding the nature of the relationship with the community would provide insights into the optimal community-based recruitment approach conducive to fostering favorable recruitment outcomes. Based on our findings, quantitative studies had a substantial risk of selection bias, study design, lack of consideration of confounders, and insufficient description of attrition. Furthermore, we observed that the majority of reviewed AD/ADRD observational and clinical trial studies provided a brief description of the recruitment strategies. As researchers strive for greater inclusivity in recruiting older adults, it’s important for the guidance around recruitment reports and metrics to reflect the needs of diverse communities and strive for equitable representation in clinical research. Furthermore, frameworks and theory-based models can help guide researchers and readers to better understand the recruitment methodology and advance community-based recruitment science.

Although the majority of reviewed clinical trials and observational studies reported the approximate geographical areas of recruitment efforts, 22% of the studies did not report this information. Geographical locations, characteristics of the research area, and whether it is a rural or urban area can provide additional insights about socio-ecological factors influencing participants’ willingness to join AD/ADRD research. Our reviewed studies suggested that there may be a lack of geographical diversity, which can also limit the generalizability of recruitment outcomes. To facilitate increasing representation of racial and ethnic minoritized community members in AD/ADRD clinical trials, we need additional evidence of protocols and community-based recruitment strategies to expound on the success of community-based recruitment methodology. Implementing pragmatic clinical trial designs that are embedded in healthcare systems (100) could facilitate increased access to racial and ethnic minoritized communities that are hardly reached by researchers but exist in the healthcare systems. Overall, evidence-based and culturally sensitive recruitment approaches could benefit the external validity of studies.

### Limitations

The current rapid review should be interpreted in the context of several limitations. Using one reviewer to ensure data extraction and quality assessment in a short turnaround time would have introduced biases. Such a design can increase susceptibility to selection bias, potential overlook of relevant studies, and incomplete evidence synthesis. The absence of quality evaluation by another reviewer is indeed a drawback. The rapid nature of the current review may have compromised rigor, potentially resulting in a trade-off between efficiency and comprehensiveness. Despite this limitation, this rapid review adhered to the systematic review protocol and followed the steps from study identification across several databases to quality assessment, all accomplished within a short timeframe.

## Conclusion

This review identified a number of recruitment strategies that utilized community-based approaches to increase the participation of minoritized communities. We found that using a multiprong recruitment approach that addresses barriers such as building trust by forming coalitions and partnerships with community-based organizations, using culturally sensitive strategies, and being receptive to the community’s needs may yield more fruitful recruitment outcomes. Such recruitment efforts can simultaneously address multiple levels of individual and institutional barriers. It is imperative that AD/ADRD clinical investigators continue their efforts to increase recruitment and engagement of racial and ethnic diversity in AD/ADRD clinical trials, as including such minoritized populations would yield more information on prospective variation in intervention outcomes. Our study reviewed AD/ADRD research studies that utilized community-based recruitment strategies to increase the representation of racial and ethnic minoritized populations with limited evidence of community engagement in recruitment in AD/ADRD clinical trials. Publishing clinical trial protocols and feasibility studies can increase evidence of community-based recruitment science. In addition, incorporating recruitment evaluation into AD/ADRD clinical trial protocols would enhance consistency in recruitment methodologies and facilitate systematic data collection on best practices.

With more evidence of community engagement as a viable recruitment strategy to increase racial and ethnic representation in AD/ADRD clinical trials, we can advance recruitment science by establishing a standardized community-based recruitment methodology.

## Electronic Supplementary Material

Supplementary material, approximately 138 KB.

## Notes

*Ethical standards:* The current systematic rapid review did not have direct access to participants of primary research studies included in the review. However, all reviewed studies reviewed reported that they had obtained written informed consent from their participants.

## References

[1] Andrieu S, Coley N, Gardette V, Subra J, Oustric S, Fournier T, et al. Representations and practices of prevention in elderly populations: investigating acceptance to participate in and adhesion to an intervention study for the prevention of Alzheimer’s disease (ACCEPT study)—the need for a multidisciplinary approach. J Nutr Health Aging. 2012;16(4):352–4. doi: 10.1007/s12603-012-0045-9. PubMed PMID: 22499457.22499457 10.1007/s12603-012-0045-9

[2] Coley N, Coniasse-Brioude D, Igier V, Fournier T, Poulain JP, Andrieu S. Disparities in the participation and adherence of older adults in lifestyle-based multidomain dementia prevention and the motivational role of perceived disease risk and intervention benefits: an observational ancillary study to a randomised controlled trial. Alzheimers Res Ther. 2021;13(1):157. Epub 20210924. doi: 10.1186/s13195-021-00904-6. PubMed PMID: 34560903; PubMed Central PMCID: PMC8464095. 34560903 10.1186/s13195-021-00904-6 PMC8464095

[3] Cocroft S, Welsh-Bohmer KA, Plassman BL, Chanti-Ketterl M, Edmonds H, Gwyther L, et al. Racially diverse participant registries to facilitate the recruitment of African Americans into presymptomatic Alzheimer’s disease studies. Alzheimer’s & dementia: the journal of the Alzheimer’s Association. 2020;16(8):1107–14. doi: 10.1002/alz.12048. PubMed PMID: 32543781. 10.1002/alz.12048 32543781

[4] Brown P, Tan AC, El-Esawi MA, Liehr T, Blanck O, Gladue DP, et al. Large expert-curated database for benchmarking document similarity detection in biomedical literature search. Database-the Journal of Biological Databases and Curation. 2019. doi: 10.1093/database/baz085. PubMed PMID: WOS:000494411700001. 10.1093/database/baz085 PMC7291946 33326193

[5] Bleakley A, Maloney EK, Harkins K, Nelson MN, Akpek E, Langbaum JB. An Elicitation Study to Understand Black, Hispanic, and Male Older Adults’ Willingness to Participate in Alzheimer’s Disease-Focused Research Registries. Journal of Alzheimers Disease. 2022;88(4):1499–509. doi: 10.3233/jad-220196. PubMed PMID: WOS:000842012100021. 10.3233/JAD-220196 PMC9720734 35811525

[6] Canevelli M, Bruno G, Grande G, Quarata F, Raganato R, Remiddi F, et al. Race reporting and disparities in clinical trials on Alzheimer’s disease: A systematic review. Neuroscience & Biobehavioral Reviews. 2019;101:122–8. doi: 10.1016/j.neubiorev.2019.03.020. 30946856 10.1016/j.neubiorev.2019.03.020

[7] Grill JD, Raman R, Ernstrom K, Aisen P, Karlawish J. Effect of study partner on the conduct of Alzheimer disease clinical trials. Neurology. 2013;80(3):282–8. Epub 20121219. doi: 10.1212/WNL.0b013e31827debfe. PubMed PMID: 23255824; PubMed Central PMCID: PMC3589183. 23255824 10.1212/WNL.0b013e31827debfe PMC3589183

[8] Kennedy RE, Cutter GR, Wang G, Schneider LS. Challenging Assumptions About African American Participation in Alzheimer Disease Trials. Am J Geriatr Psychiatry. 2017;25(10):1150–9. Epub 20170425. doi: 10.1016/j.jagp.2017.04.013. PubMed PMID: 28554539; PubMed Central PMCID: PMC5842064. 28554539 10.1016/j.jagp.2017.04.013 PMC5842064

[9] Krogstad JM. Key facts about U.S. Latinos for national Hispanic Heritage Month. In: Passel JS, editor. Noe-Bustamante, L. Pew Research Center 2022.

[10] Hispanic Population to Reach 111 Million by 2060. 2017 National Population Projections and Vintage 2017 Population Estimates: Census.gov; 2018.

[11] S C. Projections of the Size and Composition of the U.S:2014–2060. In: J O, editor. United States Census Bureau. 2015.

[12] Williams MM, Scharff DP, Mathews KJ, Hoffsuemmer JS, Jackson P, Morris JC, et al. Barriers and facilitators of African American participation in Alzheimer disease biomarker research. Alzheimer Disease and Associated Disorders. 2010;24(SUPPL. 1):S24–S9. doi: 10.1097/WAD.0b013e3181f14a14. 20711059 10.1097/WAD.0b013e3181f14a14 PMC2939138

[13] Grill JD, Karlawish J. Addressing the challenges to successful recruitment and retention in Alzheimer’s disease clinical trials. Alzheimers Res Ther. 2010;2(6):34. Epub 20101221. doi: 10.1186/alzrt58. PubMed PMID: 21172069; PubMed Central PMCID: PMC3031880. 21172069 10.1186/alzrt58 PMC3031880

[14] Gallagher-Thompson D, Singer LS, Depp C, Mausbach BT, Cardenas V, Coon DW. Effective recruitment strategies for Latino and Caucasian dementia family caregivers in intervention research. Am J Geriatr Psychiatry. 2004;12(5):484–90. doi: 10.1176/appi.ajgp.12.5.484. PubMed PMID: 15353386. 15353386 10.1176/appi.ajgp.12.5.484

[15] Raman R, Quiroz YT, Langford O, Choi J, Ritchie M, Baumgartner M, et al. Disparities by Race and Ethnicity Among Adults Recruited for a Preclinical Alzheimer Disease Trial. JAMA Netw Open. 2021;4(7):e2114364. Epub 20210701. doi: 10.1001/jamanetworkopen.2021.14364. PubMed PMID: 34228129; PubMed Central PMCID: PMC8261604. 34228129 10.1001/jamanetworkopen.2021.14364 PMC8261604

[16] Deters KD, Napolioni V, Sperling RA, Greicius MD, Mayeux R, Hohman T, et al. Amyloid PET Imaging in Self-Identified Non-Hispanic Black Participants of the Anti-Amyloid in Asymptomatic Alzheimer’s Disease (A4) Study. Neurology. 2021;96(11):e1491–e500. Epub 20210210. doi: 10.1212/wnl.0000000000011599. PubMed PMID: 33568538; PubMed Central PMCID: PMC8032379. 33568538 10.1212/WNL.0000000000011599 PMC8032379

[17] Galvin JE, Meuser TM, Boise L, Connell CM. Predictors of physician referral for patient recruitment to Alzheimer disease clinical trials. Alzheimer Dis Assoc Disord. 2009;23(4):352–6. doi: 10.1097/WAD.0b013e31819e0cac. PubMed PMID: 19561438; PubMed Central PMCID: PMC2787738. 19561438 10.1097/WAD.0b013e31819e0cac PMC2787738

[18] Watson JL, Ryan L, Silverberg N, Cahan V, Bernard MA. Obstacles and opportunities in Alzheimer’s clinical trial recruitment. Health affairs (Project Hope). 2014;33(4):574–9. doi: 10.1377/hlthaff.2013.1314. 24711317 10.1377/hlthaff.2013.1314 PMC4167360

[19] Cox CG, Davis MA, Grill JD, Roberts JS. US Adults’ Likelihood to Participate in Dementia Prevention Drug Trials: Results from the National Poll on Healthy Aging. J Prev Alzheimers Dis. 2023;10(1):34–40. doi: 10.14283/jpad.2022.86. PubMed PMID: 36641608; PubMed Central PMCID: PMC9579667. 36641608 10.14283/jpad.2022.86 PMC9579667

[20] Karlawish J, Cary MS, Rubright J, Tenhave T. How redesigning AD clinical trials might increase study partners’ willingness to participate. Neurology. 2008;71(23):1883–8. doi: 10.1212/01.wnl.0000336652.05779.ea. PubMed PMID: 19047560; PubMed Central PMCID: PMC2649726. 19047560 10.1212/01.wnl.0000336652.05779.ea PMC2649726

[21] Karlawish JH, Casarett D, Klocinski J, Sankar P. How do AD patients and their caregivers decide whether to enroll in a clinical trial? Neurology. 2001;56(6):789–92. doi: 10.1212/wnl.56.6.789. PubMed PMID: 11274319. 11274319 10.1212/wnl.56.6.789

[22] Grill JD, Karlawish J, Elashoff D, Vickrey BG. Risk disclosure and preclinical Alzheimer’s disease clinical trial enrollment. Alzheimers Dement. 2013;9(3):356–9.e1. Epub 20121108. doi: 10.1016/j.jalz.2012.03.001. PubMed PMID: 23141383; PubMed Central PMCID: PMC3572336. 23141383 10.1016/j.jalz.2012.03.001 PMC3572336

[23] Roberts JS, Connell CM, Cisewski D, Hipps YG, Demissie S, Green RC. Differences between African Americans and whites in their perceptions of Alzheimer disease. Alzheimer Dis Assoc Disord. 2003;17(1):19–26. doi: 10.1097/00002093-200301000-00003. PubMed PMID: 12621316. 12621316 10.1097/00002093-200301000-00003

[24] Alzheimer’s Disease Facts And Figures. Association As: Alzheimers Dement.2021; 2021. p. 237–406. 10.1002/alz.12328 33756057

[25] George S, Duran N, Norris K. A Systematic Review of Barriers and Facilitators to Minority Research Participation Among African Americans, Latinos, Asian Americans, and Pacific Islanders. American Journal of Public Health. 2014;104(2):e16–e31. doi: 10.2105/ajph.2013.301706. PubMed PMID: 24328648. 24328648 10.2105/AJPH.2013.301706 PMC3935672

[26] Ford ME, Siminoff LA, Pickelsimer E, Mainous AG, Smith DW, Diaz VA, et al. Unequal Burden of Disease, Unequal Participation in Clinical Trials: Solutions from African American and Latino Community Members. Health & Social Work. 2013;38(1):29–38. doi: 10.1093/hsw/hlt001. 23539894 10.1093/hsw/hlt001 PMC3943359

[27] Grill JD, Galvin JE. Facilitating Alzheimer Disease Research Recruitment. Alzheimer Disease & Associated Disorders. 2014;28(1):1–8. doi: 10.1097/wad.0000000000000016. PubMed PMID: WOS:000332086700001. 24322484 10.1097/WAD.0000000000000016 PMC3945167

[28] Otado J, Kwagyan J, Edwards D, Ukaegbu A, Rockcliffe F, Osafo N. Culturally Competent Strategies for Recruitment and Retention of African American Populations into Clinical Trials. Clin Transl Sci. 2015;8(5):460–6. Epub 20150514. doi: 10.1111/cts.12285. PubMed PMID: 25974328; PubMed Central PMCID: PMC4626379. 25974328 10.1111/cts.12285 PMC4626379

[29] Tremblay M-C, Martin DH, McComber AM, McGregor A, Macaulay AC. Understanding community-based participatory research through a social movement framework: a case study of the Kahnawake Schools Diabetes Prevention Project. BMC Public Health. 2018;18(1):487. doi: 10.1186/s12889-018-5412-y. 29650020 10.1186/s12889-018-5412-y PMC5897940

[30] Brewer LC, Jenkins S, Hayes SN, Kumbamu A, Jones C, Burke LE, et al. Community-based, cluster-randomized pilot trial of a cardiovascular mHealth intervention: Rationale, design, and baseline findings of the FAITH! Trial. American Heart Journal. 2022;247:1–14. doi: 10.1016/j.ahj.2022.01.009. 35065922 10.1016/j.ahj.2022.01.009 PMC9037298

[31] Greiner KA, Friedman DB, Adams SA, Gwede CK, Cupertino P, Engelman KK, et al. Effective Recruitment Strategies and Community-Based Participatory Research: Community Networks Program Centers’ Recruitment in Cancer Prevention Studies. Cancer Epidemiology, Biomarkers & Prevention. 2014;23(3):416–23. doi: 10.1158/1055-9965.epi-13-0760. 10.1158/1055-9965.EPI-13-0760 PMC3971731 24609851

[32] Ford ME, Havstad SL, Tilley BC. Recruiting Older African American Men to a Cancer Screening Trial (The AAMEN Project). The Gerontologist. 2003;43(1):27–35. doi: 10.1093/geront/43.1.27. 12604743 10.1093/geront/43.1.27

[33] Services UDoHaH. Principles of Community Engagement. 2nd ed. Washington, DC: US Department of Health and Human Services.; 2011.

[34] National Institutes of Health. NIH policy and guidelines on the inclusion of women and minorities as subjects in clinical research -amended, October, 2001. Available from: https://grants.nih.gov/grants/funding/women_min/guidelines_amended_10_2001.htm.

[35] Wong R, Amano T, Lin SY, Zhou Y, Morrow-Howell N. Strategies for the Recruitment and Retention of Racial/Ethnic Minorities in Alzheimer Disease and Dementia Clinical Research. Curr Alzheimer Res. 2019;16(5):458–71. doi: 10.2174/1567205016666190321161901. PubMed PMID: 30907319. 30907319 10.2174/1567205016666190321161901

[36] Gilmore-Bykovskyi AL, Jin YY, Gleason C, Flowers-Benton S, Block LM, Dilworth-Anderson P, et al. Recruitment and retention of underrepresented populations in Alzheimer’s disease research: A systematic review. Alzheimers & Dementia-Translational Research & Clinical Interventions. 2019;5(1):751–70. doi: 10.1016/j.trci.2019.09.018. PubMed PMID: WOS:000737692800079. 10.1016/j.trci.2019.09.018 PMC6944728 31921966

[37] JP H. Cochrane handbook for systematic reviews of interventions. In: Green S E, editor. London: The Cochrane Collaboration; 2011.

[38] Types of Human Subjects Research I National Institute of Dental and Craniofacial Research. www.nidcr.nih.gov. Accessed June 14, 2024. https://www.nidcr.nih.gov/research/human-subjects-research/types-of-human-subjects-research#:~:text=Back%20to%20top-

[39] Armijo-Olivo S, Stiles CR, Hagen NA, Biondo PD, Cummings GG. Assessment of study quality for systematic reviews: a comparison of the Cochrane Collaboration Risk of Bias Tool and the Effective Public Health Practice Project Quality Assessment Tool: methodological research. J Eval Clin Pract. 2012;18(1):12–8. Epub 20100804. doi: 10.1111/j.1365-2753.2010.01516.x. PubMed PMID: 20698919. 20698919 10.1111/j.1365-2753.2010.01516.x

[40] C. L. Qualitative research synthesis: methodological guidance for systematic reviewers utilizing meta-aggregation. In: Z. M, editor.: Int J Evid Based Healthc; 2015. p. 179–87. 10.1097/XEB.0000000000000062 26262565

[41] Bardach SH, Benton B, Walker C, Alfred DL, Ighodaro E, Caban-Holt A, et al. Perspectives of African American Older Adults on Brain Health “Brains Get Tired Too”. Alzheimer Disease & Associated Disorders. 2019;33(4):354–8. doi: 10.1097/wad.0000000000000335. PubMed PMID: WOS:000500026600010. 31335456 10.1097/WAD.0000000000000335 PMC7181952

[42] Cocroft S, Welsh-Bohmer KA, Plassman BL, Chanti-Ketterl M, Edmonds H, Gwyther L, et al. Racially diverse participant registries to facilitate the recruitment of African Americans into presymptomatic Alzheimer’s disease studies. Alzheimers & Dementia. 2020;16(8):1107–14. doi: 10.1002/alz.12048. PubMed PMID: WOS:000577872500001. 10.1002/alz.12048 32543781

[43] Epps F, Skemp L, Specht J. Using Culturally Informed Strategies to Enhance Recruitment of African Americans in Dementia Research: A Nurse Researcher’s Experience. Journal of Research Practice. 2015;11(1):19. PubMed PMID: WOS:000389583200003.

[44] Marquez DX, Perez A, Johnson JK, Jaldin M, Pinto J, Keiser S, et al. Increasing engagement of Hispanics/Latinos in clinical trials on Alzheimer’s disease and related dementias. Alzheimers & Dementia-Translational Research & Clinical Interventions. 2022;8(1):11. doi: 10.1002/trc2.12331. PubMed PMID: WOS:000830120200001. 10.1002/trc2.12331 PMC9322823 35910673

[45] Neugroschl J, Sewell MC, Umpierre M, Rodriguez R, Meyers L, Kranes S, et al. Elderly Latino community members make an educational video: an academic-community collaboration to promote memory evaluations. International Psychogeriatrics. 2019;31(7):989–95. doi: 10.1017/s1041610218001448. PubMed PMID: WOS:000514156700010. 30318026 10.1017/S1041610218001448 PMC6465172

[46] Parveen S, Barker S, Kaur R, Kerry F, Mitchell W, Happs A, et al. Involving minority ethnic communities and diverse experts by experience in dementia research: The Caregiving HOPE Study. Dementia (London, England). 2018;17(8):990–1000. doi: 10.1177/1471301218789558. 30373461 10.1177/1471301218789558

[47] Portacolone E, Palmer NR, Lichtenberg P, Waters CM, Hill CV, Keiser S, et al. Earning the trust of african american communities to increase representation in dementia research. Ethnicity & Disease. 2020;30:719–34. doi: 10.18865/ed.30.S2.719. PubMed PMID: WOS:000603397700003. 33250619 10.18865/ed.30.S2.719 PMC7683027

[48] Sharma RK, Teng A, Asirot MG, Taylor JO, Borson S, Turner AM. Challenges and opportunities in conducting research with older adults with dementia during COVID-19 and beyond. Journal of the American Geriatrics Society. 2022;70(5):1306–13. doi: 10.1111/jgs.17750. PubMed PMID: WOS:000770997500001. 35285942 10.1111/jgs.17750 PMC9106837

[49] Souder E, Terry TL. Use of lay educators to overcome barriers to research with Black older adults: a case study using Alzheimer’s Disease Center. Research in gerontological nursing. 2009;2(4):235–42. doi: 10.3928/19404921-20090731-04. 20077978 10.3928/19404921-20090731-04

[50] McDougall GJ, Becker H, Pituch K, Acee TW, Vaughan PW, Delville CL. Differential Benefits of Memory Training for Minority Older Adults in the SeniorWISE Study. Gerontologist. 2010;50(5):632–45. doi: 10.1093/geront/gnq017. PubMed PMID: WOS:000281962200008. 20203096 10.1093/geront/gnq017 PMC2948826

[51] Ajrouch KJ, Vega IE, Antonucci TC, Tarraf W, Webster NJ, Zahodne LB. Partnering with Middle Eastern/Arab American and Latino Immigrant Communities to Increase Participation in Alzheimer’s Disease Research. Ethn Dis. 2020;30(Suppl 2):765–74. Epub 20201119. doi: 10.18865/ed.30.S2.765. PubMed PMID: 33250623; PubMed Central PMCID: PMC7683026. 33250623 10.18865/ed.30.S2.765 PMC7683026

[52] Ashford MT, Camacho MR, Jin CS, Eichenbaum J, Ulbricht A, Alaniz R, et al. Digital culturally tailored marketing for enrolling Latino participants in a web-based registry: Baseline metrics from the Brain Health Registry. Alzheimers & Dementia. 15. doi: 10.1002/alz.12805. PubMed PMID: WOS:000863622900001. 10.1002/alz.12805 PMC10070578 36193827

[53] Bardach SH, Barber JM, Schmitt FA, Van Eldik LJ, Boggess MB, Yarbrough M, et al. The Effectiveness of Community-based Outreach Events for the Promotion of African American Research Participation. Alzheimer Dis Assoc Disord. 2020;34(4):344–9. doi: 10.1097/wad.0000000000000404. PubMed PMID: 32809985; PubMed Central PMCID: PMC7677178. 32809985 10.1097/WAD.0000000000000404 PMC7677178

[54] Barnes LL, Shah RC, Aggarwal NT, Bennett DA, Schneider JA. The Minority Aging Research Study: Ongoing Efforts to Obtain Brain Donation in African Americans without Dementia. Current Alzheimer Research. 2012;9(6):734–45. doi: 10.2174/156720512801322627. PubMed PMID: WOS:000308864500012. 22471868 10.2174/156720512801322627 PMC3409294

[55] Boyd AD, Railey AF, Kirkpatrick AW, Hsu YC, Muller C, Buchwald D. Communication about Alzheimer’s disease and research among American Indians and Alaska Natives. Alzheimers & Dementia-Translational Research & Clinical Interventions. 2022;8(1):8. doi: 10.1002/trc2.12302. PubMed PMID: WOS:000793258400001. 10.1002/trc2.12302 PMC9093044 35592690

[56] Chao SZ, Lai NB, Tse MM, Ho RJ, Kong JP, Matthews BR, et al. Recruitment of Chinese American Elders into Dementia Research: The UCSF ADRC Experience. Gerontologist. 2011;51:S125–S33. doi: 10.1093/geront/gnr033. PubMed PMID: WOS:000290611300013. 21565814 10.1093/geront/gnr033 PMC3092979

[57] DeCaro R, O’Connor MK, DiTerlizzi C, Sekyi-Appiah N, Polk J, Budson AE. Educating students while recruiting underrepresented populations for Alzheimer’s disease research: the Student Ambassador Program. Bmc Medical Education. 2022;22(1). doi: 10.1186/s12909-022-03749-1. PubMed PMID: WOS:000864118600001. 10.1186/s12909-022-03749-1 PMC9533970 36199070

[58] Etkin CD, Farran CJ, Barnes LL, Shah RC. Recruitment and enrollment of caregivers for a lifestyle physical activity clinical trial. Research in Nursing & Health. 2012;35(1):70–81. doi: 10.1002/nur.20466. PubMed PMID: WOS:000298672200007. 22083931 10.1002/nur.20466 PMC3729020

[59] Fritsch T, Adams KB, Redd D, Sias T, Herrup K. Use of live theater to increase minority participation in Alzheimer disease research. Alzheimer Dis Assoc Disord. 2006;20(2):105–11. doi: 10.1097/01.wad.0000213806.66811.ea. PubMed PMID: 16772746. 16772746 10.1097/01.wad.0000213806.66811.ea

[60] Gallagher-Thompson D, Rabinowitz Y, Tang PCY, Tse C, Kwo E, Hsu S, et al. Recruiting Chinese Americans for dementia caregiver intervention research: Suggestions for success. American Journal of Geriatric Psychiatry. 2006;14(8):676–83. doi: 10.1097/01.JGP.0000221234.65585.f9. PubMed PMID: WOS:000239205500006. 10.1097/01.JGP.0000221234.65585.f9 16861372

[61] Han HR, Choi S, Wang J, Lee HB. Pilot testing of a dementia literacy intervention for Korean American elders with dementia and their caregivers. J Clin Transl Res. 2021;7(6):712–6. Epub 20211030. PubMed PMID: 34901516; PubMed Central PMCID: PMC8654362. 34901516 PMC8654362

[62] Hinton L, Carter K, Reed BR, Beckett L, Lara E, DeCarli C, et al. Recruitment of a Community-based Cohort for Research on Diversity and Risk of Dementia. Alzheimer Disease & Associated Disorders. 2010;24(3):234–41. doi: 10.1097/WAD.0b013e3181c1ee01. PubMed PMID: WOS:000281310200004. 20625273 10.1097/WAD.0b013e3181c1ee01 PMC2946798

[63] Howell JC, Soyinka O, Parker M, Jarrett TL, Roberts DL, Dorbin CD, et al. Knowledge and Attitudes in Alzheimer’s Disease in a Cohort of Older African Americans and Caucasians. American Journal of Alzheimers Disease and Other Dementias. 2016;31(4):361–7. doi: 10.1177/1533317515619037. PubMed PMID: WOS:000376305200008. 10.1177/1533317515619037 PMC4867248 26646115

[64] Li C, Neugroschl J, Umpierre M, Martin J, Huang QY, Zeng XY, et al. Recruiting US Chinese Elders Into Clinical Research for Dementia. Alzheimer Disease & Associated Disorders. 2016;30(4):345–7. doi: 10.1097/wad.0000000000000162. PubMed PMID: WOS:000389258200009. 27819841 10.1097/WAD.0000000000000162 PMC5119898

[65] Lingler JH, Ren D, Tamres LK, Knox ML, Mbawuike U, Williams IC, et al. Mechanisms by which Cultural-Centric Narrative Influences Interest in ADRD Research Among African American Adults. The Gerontologist. 2022. doi: 10.1093/geront/gnac179. 10.1093/geront/gnac179 PMC10353039 36544399

[66] Meyer OL, Sun MX, Do T, Ho JN, Dinh BT, Nguyen S, et al. Community-Engaged Research with Vietnamese Americans to Pilot-Test a Dementia Caregiver Intervention. Journal of Cross-Cultural Gerontology. 2020;35(4):479–92. doi: 10.1007/s10823-020-09410-y. PubMed PMID: WOS:000561512000001. 32821996 10.1007/s10823-020-09410-y PMC7584753

[67] Milani SA, Swain M, Otufowora A, Cottler LB, Striley CW. Willingness to Participate in Health Research Among Community-Dwelling Middle-Aged and Older Adults: Does Race/Ethnicity Matter? Journal of Racial and Ethnic Health Disparities. 2021;8(3):773–82. doi: 10.1007/s40615-020-00839-y. PubMed PMID: WOS:000560312500004. 32808194 10.1007/s40615-020-00839-y PMC7431111

[68] Overman AA, Robbins RE. Game-Based Community Cognitive Health Intervention for Minority and Lower Socioeconomic Status Older Adults: A Feasibility Pilot Study. Games for Health Journal. 2014;3(5):303–10. doi: 10.1089/g4h.2014.0052. PubMed PMID: WOS:000363384100006. 26192485 10.1089/g4h.2014.0052

[69] Parker LJ, Gaugler JE, Gitlin LN. Use of Critical Race Theory to Inform the Recruitment of Black/African American Alzheimer’s Disease Caregivers into Community-Based Research. The Gerontologist. 2022;62(5):742–50. doi: 10.1093/geront/gnac001. 34999789 10.1093/geront/gnac001 PMC9154262

[70] Perales-Puchalt J, Shaw A, McGee JL, Moore WT, Hinton L, Resendez J, et al. Preliminary Efficacy of a Recruitment Educational Strategy on Alzheimer’s Disease Knowledge, Research Participation Attitudes, and Enrollment Among Hispanics. Hisp Health Care Int. 2020;18(3):144–9. Epub 20191215. doi: 10.1177/1540415319893238. PubMed PMID: 31840539; PubMed Central PMCID: PMC7293919. 31840539 10.1177/1540415319893238 PMC7293919

[71] Romero HR, Welsh-Bohmer KA, Gwyther LP, Edmonds HL, Plassman BL, Germain CM, et al. Community engagement in diverse populations for Alzheimer disease prevention trials. Alzheimer Dis Assoc Disord. 2014;28(3):269–74. doi: 10.1097/wad.0000000000000029. PubMed PMID: 24614272; PubMed Central PMCID: PMC4139415. 24614272 10.1097/WAD.0000000000000029 PMC4139415

[72] Samus QM, Amjad H, Johnston D, Black BS, Bartels SJ, Lyketsos CG. A Multipronged, Adaptive Approach for the Recruitment of Diverse Community-Residing Elders with Memory Impairment: The MIND at Home Experience. American Journal of Geriatric Psychiatry. 2015;23(7):698–708. doi: 10.1016/j.jagp.2015.01.005. PubMed PMID: WOS:000355926200006. 10.1016/j.jagp.2015.01.005 PMC5226267 25771267

[73] Shaw AR, Perales-Puchalt J, Moore T, Weatherspoon P, Robinson M, Hill CV, et al. Recruitment of Older African Americans in Alzheimer’s Disease Clinical Trials Using a Community Education Approach. Jpad-Journal of Prevention of Alzheimers Disease. 2022;9(4):672–8. doi: 10.14283/jpad.2022.82. PubMed PMID: WOS:000860368400002. 10.14283/jpad.2022.82 PMC9514712 36281671

[74] Ta Park VM, Meyer OL, Tsoh JY, Kanaya AM, Tzuang M, Nam B, et al. The Collaborative Approach for Asian Americans and Pacific Islanders Research and Education (CARE): A recruitment registry for Alzheimer’s disease and related dementias, aging, and caregiver-related research. Alzheimers Dement. 2023;19(2):433–43. Epub 20220414. doi: 10.1002/alz.12667. PubMed PMID: 35420258; PubMed Central PMCID: PMC9562598. 35420258 10.1002/alz.12667 PMC9562598

[75] Weiner MW, Veitch DP, Miller MJ, Aisen PS, Albala B, Beckett LA, et al. Increasing participant diversity in AD research: Plans for digital screening, blood testing, and a community-engaged approach in the Alzheimer’s Disease Neuroimaging Initiative 4. Alzheimers Dement. 2023;19(1):307–17. Epub 20221009. doi: 10.1002/alz.12797. PubMed PMID: 36209495; PubMed Central PMCID: PMC10042173. 36209495 10.1002/alz.12797 PMC10042173

[76] Wiese LK, Williams IC, Schoenberg NE, Galvin JE, Lingler J. Overcoming the COVID-19 Pandemic for Dementia Research: Engaging Rural, Older, Racially and Ethnically Diverse Church Attendees in Remote Recruitment, Intervention and Assessment. Gerontology and Geriatric Medicine. 2021;7. doi: 10.1177/23337214211058919. PubMed PMID: WOS:000721360800001. 10.1177/23337214211058919 PMC8609097 34825019

[77] Zhou Y, Elashoff D, Kremen S, Teng E, Karlawish J, Grill JD. African Americans are less likely to enroll in preclinical Alzheimer’s disease clinical trials. Alzheimer’s & dementia (New York, N Y). 2016;3(1):57–64. doi: 10.1016/j.trci.2016.09.004. PubMed PMID: 29067319. 10.1016/j.trci.2016.09.004 PMC5651355 29067319

[78] O’Bryant SE, Johnson L, Balldin V, Edwards M, Barber R, Williams B, et al. Characterization of Mexican Americans with mild cognitive impairment and Alzheimer’s disease. J Alzheimers Dis. 2013;33(2):373–9. doi: 10.3233/jad-2012-121420. PubMed PMID: 22976076; PubMed Central PMCID: PMC3524411. 22976076 10.3233/JAD-2012-121420 PMC3524411

[79] Bachman DL, Stuckey M, Ebeling M, Wagner MT, Evans WJ, Hirth V, et al. Establishment of a predominantly African-American cohort for the study of Alzheimer’s disease: the South Carolina Alzheimer’s disease clinical core. Dementia and geriatric cognitive disorders. 2009;27(4):329–36. doi: 10.1159/000207446. 19276625 10.1159/000207446

[80] Ballard EL, Nash F, Raiford K, Harrell LE. Recruitment of black elderly for clinical research studies of dementia - the cerad experience. Gerontologist. 1993;33(4):561–5. doi: 10.1093/geront/33.4.561. PubMed PMID: WOS:A1993LQ87400021. 8375688 10.1093/geront/33.4.561

[81] Gauthier MA, Clarke WP. Gaining and sustaining minority participation in longitudinal research projects. Alzheimer Disease & Associated Disorders. 1999;13:S29–S33. doi: 10.1097/00002093-199904001-00008. PubMed PMID: WOS:000084471700008. 10369515 10.1097/00002093-199904001-00008

[82] Rexroth DF, Friedland RP. Lessons learned regarding recruitment to the national African American Alzheimer disease health literacy program. Alzheimer Disease and Associated Disorders. 2010;24(SUPPL. 1):S54–S7. doi: 10.1097/WAD.0b013e3181f14b22. 22720322

[83] Williams MM, Meisel MM, Williams J, Morris JC. An Interdisciplinary Outreach Model of African American Recruitment for Alzheimer’s Disease Research. Gerontologist. 2011;51:S134–S41. doi: 10.1093/geront/gnq098. PubMed PMID: WOS:000290611300014. 21173436 10.1093/geront/gnq098 PMC3092972

[84] Nkimbeng M, Rosebush CE, Akosah KO, Yam H, Russell WN, Bustamante G, et al. The Immigrant Memory Collaborative: A Community-University Partnership to Assess African Immigrant Families’ Experiences with Dementia. International Journal of Environmental Research and Public Health. 2022;19(7):19. doi: 10.3390/ijerph19074075. PubMed PMID: WOS:000783113200001. 10.3390/ijerph19074075 PMC8997896 35409758

[85] Sun F, Gao X, Shen H, Burnette D. Levels and correlates of knowledge about Alzheimer’s disease among older Chinese Americans. J Cross Cult Gerontol. 2014;29(2):173–83. doi: 10.1007/s10823-014-9229-6. PubMed PMID: 24728621. 24728621 10.1007/s10823-014-9229-6

[86] Withers M, Sayegh P, Rodriguez-Agudelo Y, Ernstrom K, Raman R, Montoya L, et al. A mixed-methods study of cultural beliefs about dementia and genetic testing among Mexicans and Mexican-Americans at-risk for autosomal dominant Alzheimer’s disease. Journal of Genetic Counseling. 2019;28(5):921–32. doi: 10.1002/jgc4.1133. PubMed PMID: WOS:000489240200001. 31207006 10.1002/jgc4.1133 PMC7500864

[87] Ashford MT, Zhu D, Bride J, McLean E, Aaronson A, Conti C, et al. Understanding Online Registry Facilitators and Barriers Experienced by Black Brain Health Registry Participants: The Community Engaged Digital Alzheimer’s Research (CEDAR) Study. Jpad-Journal of Prevention of Alzheimers Disease. doi: 10.14283/jpad.2023.25. PubMed PMID: WOS:000946893400001. 10.14283/jpad.2023.25 PMC10395260 37357297

[88] Israel BA, Eng E, Schulz AJ, Parker EA. Introduction to methods in community-based participatory research for health. Methods in community-based participatory research for health. 2005;3:26.

[89] Bartsch DAP, Rodgers VKM, Strong DELMS. Outcomes of Senior Reach Gatekeeper Referrals: Comparison of the Spokane Gatekeeper Program, Colorado Senior Reach, and Mid-Kansas Senior Outreach. Care Management Journals. 2013;14(1):11–20. doi: 10.1891/1521-0987.14.1.11. PubMed PMID: 1346648143; 23721039. 23721039 10.1891/1521-0987.14.1.11

[90] Fishbein M, Ajzen I. Predicting and Changing Behavior: The Reasoned Action Approach: Psychology Press; 2009.

[91] Kleinman A, Benson P. Anthropology in the Clinic: The Problem of Cultural Competency and How to Fix It. PLOS Medicine. 2006;3(10):e294. doi: 10.1371/journal.pmed.0030294. 17076546 10.1371/journal.pmed.0030294 PMC1621088

[92] Leventhal H, Brissette I. The common sense model of self-regulation of health and illness In: Cameron LD, Leventhal H, eds. The self-regulation of health and illness behavior. London: Routledge; 2003.

[93] Arnstein SR. A ladder of citizen participation: Journal of the American Institute of Planners; 1969.

[94] Morrow E, Ross F, Grocott P, Bennett J. A model and measure for quality service user involvement in health research. International Journal of Consumer Studies. 2010;34(5):532–9. doi: 10.1111/j.1470-6431.2010.00901.x.

[95] McDougall GJ, Jr., Simpson G, Friend ML. Strategies for research recruitment and retention of older adults of racial and ethnic minorities. Journal of gerontological nursing. 2015;41(5):14–5. doi: 10.3928/00989134-20150325-01. 25849063 10.3928/00989134-20150325-01 PMC6415923

[96] Wiese LK, Williams CL, Tappen R, Newman D, Rosselli M. Assessment of Basic Knowledge About Alzheimer’s Disease Among Older Rural Residents: A Pilot Test of a New Measure. J Nurs Meas. 2017 Dec 1;25(3):519–548. doi: 10.1891/1061-3749.25.3.519. PMID: 29268833. 29268833 10.1891/1061-3749.25.3.519

[97] Carpenter BD, Balsis S, Otilingam PG, Hanson PK, Gatz M. The Alzheimer’s Disease Knowledge Scale: development and psychometric properties. Gerontologist. 2009 Apr;49(2):236–47. doi: 10.1093/geront/gnp023. Epub 2009 Mar 25. PMID: 19363018; PMCID: PMC2667675. 19363018 10.1093/geront/gnp023 PMC2667675

[98] Roberts JS, Connell CM. Illness representations among first-degree relatives of people with Alzheimer disease. Alzheimer Dis Assoc Disord. 2000 Jul–Sep;14(3):129–136, Discussion 127–8. doi: 10.1097/00002093-200007000-00003. PMID: 10994653. 10994653 10.1097/00002093-200007000-00003

[99] Annear MJ, Toye CM, Eccleston CE, McInerney FJ, Elliott KE, Tranter BK, Hartley T, Robinson AL. Dementia Knowledge Assessment Scale: Development and Preliminary Psychometric Properties. J Am Geriatr Soc. 2015 Nov;63(11):2375–81. doi: 10.1111/jgs.13707. Epub 2015 Oct 27. PMID: 26503020. 26503020 10.1111/jgs.13707

[100] Quinones AR, Mitchell SL, Jackson JD, Aranda MP, Dilworth-Anderson P, McCarthy EP, et al. Achieving Health Equity in Embedded Pragmatic Trials for People Living with Dementia and Their Family Caregivers. Journal of the American Geriatrics Society (JAGS). 2020;68(2):S8–S13. doi: 10.1111/jgs.16614. 10.1111/jgs.16614 PMC7422698 32589281

