# Supplementary material for: Examining the Role of Community Engagement in Enhancing the Participation of Racial and Ethnic Minoritized Communities in Alzheimer’s Disease Clinical Trials; A Rapid Review
Source: J Prev Alzheimers Dis. 2024 Aug 9;11(6):1647–72. doi: 10.14283/jpad.2024.149 (PMC11573826; doi:10.14283/jpad.2024.149)
Supplement: Supplementary file 1 — Supplementary material, approximately 138 KB. [file 42414_2024_149_MOESM1_ESM.docx]

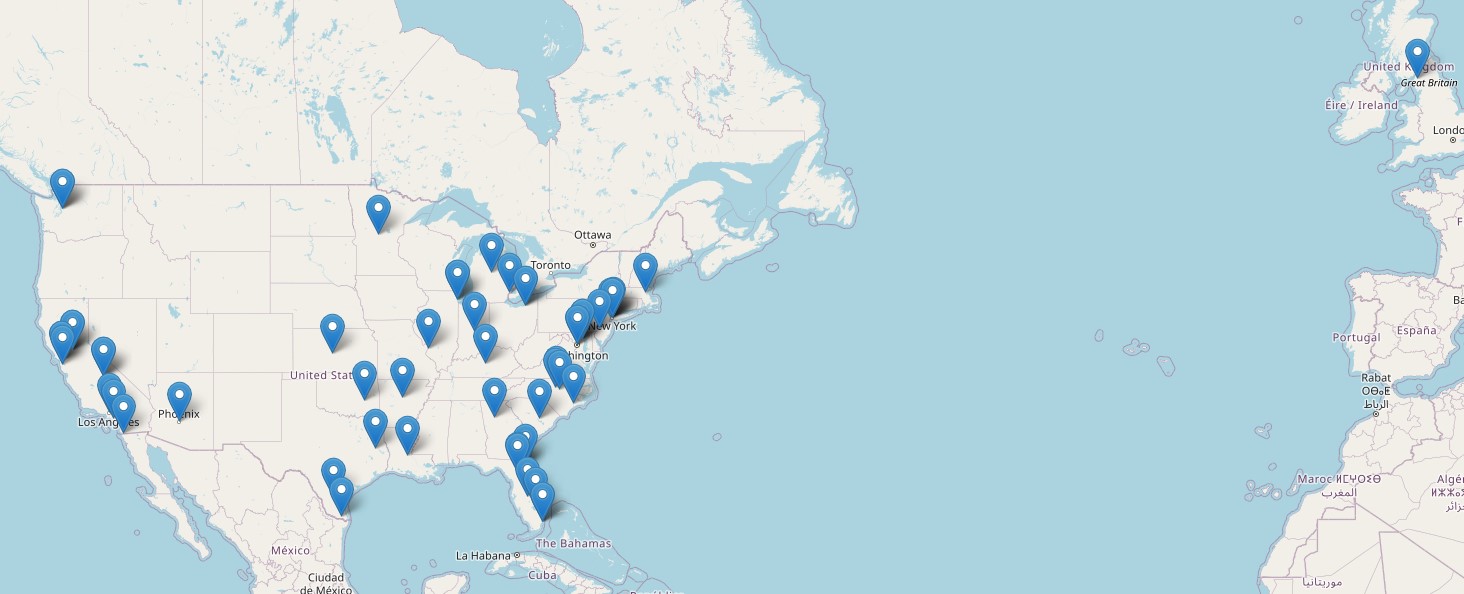


| **Author** | **Geographic area** | **Author** | **Geographic area** | **Author** | **Geographic area** | **Author** | **Geographic area** | **Author** | **Geographic area** |
| --- | --- | --- | --- | --- | --- | --- | --- | --- | --- |
| **Fritsch 2006** | Cleveland | **Bardach 2020** | Kentucky | **Garza,2020** | Lower Rio Grande Valley | **Marquez 2022** | California | **Nkimbeng 2022** | Minnesota |
| **Etkin 2012** | Chicago | **Gallagher-Thompson, 2006** | San Francisco | **Gauthier, 1999** | Boston area | **McDougall 2015** | Central Texas | **Neugroschl 2019** | East Harlem, New York |
| **Epps 2015** | Louisiana | **Gallagher-Thompson, 2004** | San Francisco | **Lingler 2022** | Pittsburg area | **Meyer2020** | Sacramento area | **Portacolone 2020** | Detroit |
| **Ajrouch 2020** | Michigan | **Sharma 2022** | Seattle | **Shaw 2022** | Kansas | **Souder 2009** | Arkansas | **Bleakley 2022** | Philadelphia |
| **Sun 2014** | Phoenix | **Wiese 2021** | Lake Okeechobee | **Withers 2019** | Tijuana | **Bachman 2009** | South Carolina | **Han, 2021** | Washington D.C. |
| **TaPark 2023** | California, UC Davis, UC Irvine, | **Williams 2011** | St. Louis | **Ashford 2021** | California | **Han, 2021** | Baltimore | **Howell 2016** | Atlanta |
| **Rexroth 2010** | Chicago,  Cleveland,  Indianapolis,  Los Angeles,  Newark,  Washington D.C.,  Durham, Chapel Hill, and Raleigh |  |  |  |  |  |  |  |  |
